# Maternal Factors Are Associated with the Expression of Placental Genes Involved in Amino Acid Metabolism and Transport

**DOI:** 10.1371/journal.pone.0143653

**Published:** 2015-12-14

**Authors:** Pricilla E. Day, Georgia Ntani, Sarah R. Crozier, Pam A. Mahon, Hazel M. Inskip, Cyrus Cooper, Nicholas C. Harvey, Keith M. Godfrey, Mark A. Hanson, Rohan M. Lewis, Jane K. Cleal

**Affiliations:** 1 Institute of Developmental Sciences, University of Southampton, Tremona Road, Southampton, SO16 6YD, United Kingdom; 2 MRC Lifecourse Epidemiology Unit, University of Southampton, Tremona Road, Southampton, SO16 6YD, United Kingdom; 3 NIHR Southampton Biomedical Research Centre, University of Southampton and University Hospital Southampton NHS Foundation Trust, Tremona Road, Southampton, SO16 6YD, United Kingdom; 4 NIHR Musculoskeletal Biomedical Research Unit, University of Oxford, Nuffield Orthopedic Centre, Headington, Oxford, OX3 7HE, United Kingdom; 5 Institute for Life Sciences, University of Southampton, Southampton, SO17 1BJ, United Kingdom; Instituto de Ciências Biomédicas / Universidade de São Paulo—USP, BRAZIL

## Abstract

**Introduction:**

Maternal environment and lifestyle factors may modify placental function to match the mother’s capacity to support the demands of fetal growth. Much remains to be understood about maternal influences on placental metabolic and amino acid transporter gene expression. We investigated the influences of maternal lifestyle and body composition (e.g. fat and muscle content) on a selection of metabolic and amino acid transporter genes and their associations with fetal growth.

**Methods:**

RNA was extracted from 102 term Southampton Women’s Survey placental samples. Expression of nine metabolic, seven exchange, eight accumulative and three facilitated transporter genes was analyzed using quantitative real-time PCR.

**Results:**

Increased placental *LAT2* (p = 0.01), *y* ^*+*^ *LAT2* (p = 0.03), aspartate aminotransferase 2 (p = 0.02) and decreased aspartate aminotransferase 1 (p = 0.04) mRNA expression associated with pre-pregnancy maternal smoking. Placental mRNA expression of *TAT1* (p = 0.01), *ASCT1* (p = 0.03), mitochondrial branched chain aminotransferase (p = 0.02) and glutamine synthetase (p = 0.05) was positively associated with maternal strenuous exercise. Increased glutamine synthetase mRNA expression (r = 0.20, p = 0.05) associated with higher maternal diet quality (prudent dietary pattern) pre-pregnancy. Lower *LAT4* (r = -0.25, p = 0.05) and aspartate aminotransferase 2 mRNA expression (r = -0.28, p = 0.01) associated with higher early pregnancy diet quality. Lower placental *ASCT1* mRNA expression associated with measures of increased maternal fat mass, including pre-pregnancy BMI (r = -0.26, p = 0.01). Lower placental mRNA expression of alanine aminotransferase 2 associated with greater neonatal adiposity, for example neonatal subscapular skinfold thickness (r = -0.33, p = 0.001).

**Conclusion:**

A number of maternal influences have been linked with outcomes in childhood, independently of neonatal size; our finding of associations between placental expression of transporter and metabolic genes and maternal smoking, physical activity and diet raises the possibility that their effects are mediated in part through alterations in placental function. The observed changes in placental gene expression in relation to modifiable maternal factors are important as they could form part of interventions aimed at maintaining a healthy lifestyle for the mother and for optimal fetal development.

## Introduction

Placental function can be altered by both maternal and fetal environmental factors[1] Maternal factors may reflect the mother’s capacity to support fetal growth, while fetal factors may reflect the fetal growth trajectory and metabolic demand[1]. The placental response to these signals determines partitioning of nutrients between the mother and fetus, determining the nutrient availability to support fetal development. Understanding maternal influences on placental function is important as fetal growth influences the risk of developing chronic diseases in later life [2]. While key regulatory pathways in the placenta, such as mTOR, are known to respond to the intrauterine environment, many aspects of the regulation of placental function remain to be determined [3].

Maternal hormones [4–6] and plasma nutrient levels are known to affect placental amino acid transporter levels by discernible mechanisms [7]. Other factors, such as maternal body composition, have been associated with changes in placental function, although the mechanism remains unclear [8,9]. Body composition may be a proxy for maternal nutrition or other lifestyle factors over a longer timeframe. Maternal lifestyle before and during pregnancy could also influence placental function. For example, maternal nutrient restriction throughout pregnancy or periods of fasting such as during Ramadan can influence placental growth [10,11]. Other factors such as exercise, drug or alcohol consumption and smoking have also been shown to influence placental development and function [12–14]. These are potentially modifiable lifestyle factors and as such could form part of interventions aimed at maintaining a healthy lifestyle for the mother and optimal development for her fetus.

Placental amino acid transfer is one aspect of placental function that can be regulated by both maternal and fetal signals. As transfer is determined by amino acid metabolism and transport, altered placental expression of the transporters and metabolic enzymes involved may act as markers of placental responses to the maternal environment[4,7,15]. Changes in placental gene expression could also be validated to become biomarkers for fetal growth parameters, postnatal growth trajectory and potentially disease risk in adulthood. Although their expression would need to be considered as part of a bigger gene network. In the present study we measured the expression of representative transporters from the three classes of amino acid transporters in the human placenta; accumulative transporters, amino acid exchangers and facilitated transporters. Accumulative transporters mediate net uptake of specific maternal amino acids (e.g. SNATs/system A) or uptake of fetal glutamate for placental glutamine synthesis (e.g. EAATs). We measured all system A members known to be expressed in human placenta as system A activity has previously been associated with maternal body composition [8]. Amino acid exchange transporters (e.g. LAT, y^+^LAT and ASC) drive uptake and transfer of other amino acids using the gradients built up by accumulative transporters. We were also interested in the facilitated transporters TAT1, LAT3 and LAT4 as they provide net amino acid transport to the fetus and their gene expression in human placenta is associated with measures of fetal growth[15]. In terms of metabolism, we focused on gene expression of enzymes involved in glutamate metabolism (for example *GLUL* and *GLUD*) as this may influence the transport and metabolism of other amino acids as well as nitrogen flux and cell growth [16].

This exploratory study aimed to identify relationships between maternal factors, fetal growth parameters and changes in placental gene expression. We used placentas from well characterised pregnancies in the Southampton Women’s Survey (SWS). The SWS is a large prospective study investigating how a mother’s diet and lifestyle influence the development of her offspring [17].

## Methods

The study was conducted according to the guidelines in the Declaration of Helsinki, and the Southampton and South West Hampshire Research Ethics Committee approved all procedures (276/97, 307/97). Written informed consent was obtained from all participating women and by parents or guardians with parental responsibility on behalf of their children.

### Maternal measurements

We used data and samples from the SWS, a cohort study of 3,158 pregnancies with information collected from the mothers before conception [17]. Non-pregnant women aged 20–34 years were recruited via their General Practitioners; assessments of lifestyle, diet and anthropometry were performed at study entry and in early (11 weeks) and late (34 weeks) gestation in those who became pregnant.

At the initial pre-pregnancy interview a dichotomous variable was derived indicating whether mothers had stated they had taken strenuous exercise (for example cycling or jogging) over the previous three months. At this time a dichotomous variable was also derived based on whether the woman perceived herself to have a faster or slower than normal walking speed. Educational attainment was defined according to the woman's highest academic qualification: examinations for General Certificate of Secondary Education (GCSEs), Advanced level (A-levels), Higher National Diploma (HNDs) and degrees thereafter. Social class was defined according to the woman's employment or to that of the head of the household and a deprivation score calculated for the address at which she lived [18]. The women’s own reported birth weight, current weight and BMI was also obtained.

Maternal smoking before and during pregnancy was assessed by questionnaire and a dichotomous variable was derived for each time point. The sum of four skinfold thickness measurements (triceps, biceps, subscapular and supra-iliac) were made on the non-dominant side to the nearest 0.1 mm in triplicate using Harpenden skinfold calipers. These were used to estimate fat mass by the method of Durnin and Womersley [19]. A tape measure was used to measure calf circumference and mid-upper arm circumference [20] from which arm muscle area was derived [21]. Diet was assessed using validated food frequency questionnaires [22]. Principal component analysis was used to summarise the dietary data. Women who had high scores on the first principal component ate diets generally consistent with healthy eating recommendations; this was termed the prudent diet score. Women who had high scores on the second principal component ate generally higher energy diets; this was termed the high-energy diet score. These scores were standardised to a mean of zero and a standard deviation of one [22]. Maternal weight gain between the initial interview and 34 weeks pregnancy was calculated.

### Placental samples

Placentas were collected from term SWS pregnancies within 30 minutes of delivery. Placental weight was measured after removing blood clots, cutting the umbilical cord flush with its insertion into the placenta, trimming away surrounding membranes and removing the amnion from the basal plate. Five villous tissue samples were selected from each placenta using a stratified random sampling method (to ensure that the selected samples were representative of the placenta as a whole); the maternal decidua was cut off of each sample. Samples were snap frozen in liquid nitrogen and stored at -80°C. For this study, a cohort of 102 placentae was selected from 300 collected in total based on availability of neonatal data.

### Fetal and neonatal measurements

Gestational age was calculated from the combination of mother’s last menstrual period date and early ultrasound data in comparison to a reference group of pregnancies of known gestational age in relation to size. Measures of fetal size were determined at 19 and 34 weeks of gestation using a high resolution ultrasound system (Acuson 128 XP, Aspen and Sequoia) calibrated to 1540 m/s. Experienced research ultrasonographers used standardised anatomical landmarks to measure head circumference, abdominal circumference and femur length [23]. The coefficient of variation for linear femur length measurements was 0.6% at 19 weeks and 0.4% at 34 weeks [24]. For elliptical head and abdominal circumference measurements, the values were 4.4% at 19 and 3.2% at 34 weeks. Shortly after delivery, research midwives measured fetal weight, head, abdominal and mid-upper arm circumference, crown–heel length, and crown–rump length. Royston’s method was used to derive z-scores for ultrasound measurements of size and conditional growth (between pairs of time points) [25,26]. The method corrects for variation in age at measurement. Conditional Z-scores for growth were derived to account for regression to the mean.

### RNA extraction and cDNA synthesis

For each placenta, the five samples were pooled and powdered in a frozen tissue press. Total RNA was extracted from 30 mg powdered placental tissue using the Rneasy fibrous tissue RNA isolation mini kit (Qiagen, UK) according to the manufacturer’s instructions. The integrity of total RNA was confirmed by agarose gel electrophoresis. Total RNA (0.2 μg) was reverse transcribed with 0.5 μg random hexamer primer, 200 units (u) M-MLV reverse transcriptase, 25 u recombinant RNasin ribonuclease inhibitor and 0.5 mM each of dATP, dCTP, dGTP and dTTP in a final reaction volume of 25 μl in 1x MMLV reaction buffer (Promega, Wisconsin, USA). Each of the 102 samples was reverse transcribed individually at the same time to reduce variation.

### Gene expression

The genes measured in this study along with primer and probe details are listed in Table 1 and Table 2. mRNA levels were measured using quantitative real-time PCR using a Roche Light-Cycler-480. For Roche Universal Probe Library probes the cycle parameters were 95°C for 10 min, followed by 40 cycles of 95°C for 15 s and 60°C for 1 min. For Primer Design Perfect Probes the cycle parameters were 95°C for 10 min, followed by 40 cycles of 95°C for 10 s and 60°C and 72°C for 15 s. Intra-assay CV’s for each gene were 5–8%. Each of the 102 samples was run on the same plate in triplicate. All mRNA levels are presented relative to the geometric mean of the three control genes, tyrosine 3-monooxygenase/tryptophan 5-monooxygenase activation protein, zeta polypeptide (*YWHAZ*), ubiquitin C (*UBC*) and topoisomerase (*TOP1*) [27].

**Table 1:** Information on amino acid transporter genes, primers and probes.

Amino acid transporter	Gene	Gene ID	Genebank accession no.	Primers	Roche universal probe library no.
LAT1	SLC7A5	8140	NM_003486.5	F-5′-gtggaaaaacaagcccaagt-3’R-5′-gcatgagcttctgacacagg-3’	25
LAT2	SLC7A8	23428	NM_182728.1NM_012244.2	F-5′-ttgccaatgtcgcttatgtc-3’R-5′-ggagcttctctccaaaagtcac-3’	17
ASCT1	SLC1A4	6509	NM_003038.2	F-5′-tttgcgacagcatttgctac-3’R-5′-gcacttcatcatagagggaagg-3’	78
ASCT2	SLC1A5	6510	NM_005628.2NM_001145145.1	F-5′-gaggaatatcaccggaacca-3’R-5′-aggatgttcatcccctcca-3’	43
y^+^LAT1	SLC7A7	9056	NM_001126106.1NM_003982.3	F-5′-acactgccgtgagaacctg-3’R-5′-aggagaggaaacccttcacc-3’	72
y^+^LAT2	SLC7A6	9057	NM_001076785.1NM_003983.4	F-5′-gctgtgatcccccatacct-3’R-5′-ggcacagttcacaaatgtcag-3’	66
4F2HC	SLC3A2	6520	NM_001012661.1	F-5′-tggttctccactcaggttga-3’R-5′-cagccaaaactccagagcat-3’	49
EAAT1	SLC1A3	6507	NM_004172.4	F-5′-ttgaactgaacttcggacaaatta-3’R-5′-attccagctgccccaatact-3’	76
EAAT2	SLC1A2	6506	NM_004171.3	F-5′-aaaatgctcattctccctctaatc-3’R-5′-gccactagccttagcatcca-3’	78
EAAT3	SLC1A1	6505	NM_004170.4	F-5′-agttgaatgacctggacttgg-3’R-5′-gcagatgtggccgtgatac-3’	9
EAAT4	SLC1A6	6511	NM_005071.1	F-5′-tgcagatgctggtgttacct-3’R-5′-gttgtccagggatgccata-3’	19
EAAT5	SLC1A7	6512	NM_006671.4	F-5′-cgcccaggtcaacaactac-3’R-5′-gctgcagtggctgtgatact-5’	9
SNAT1	SLC38A1	81539	NM_030674.3NM_001077484.1	F-5′-attttgggactcgcctttg-3’R-5′-agcaatgtcactgaagtcaaaagt-3’	47
SNAT2	SLC38A2	54407	NM_018976.3	F-5′-cctatgaaatctgtacaaaagattgg-3’F-5′-ttgtgtacccaatccaaaacaa-3’	9
SNAT4	SLC38A4	55089	NM_018018.4NM_001143824.1	F-5′-tgttctggtcatccttgtgc-3’R-5′-aaaactgctggaagaataaaaatcag-3’	29
TAT1	SLC16A10	117247	NM_018593.4	F-5′-ggtgtgaagaaggtttatctacagg-3′R-5′-agggccccaaagatgcta-3′	6
LAT3	SLC43A1	8501	NM_003627.5NM_001198810.1	F-5′-gccctcatgattggctctta-3’R-5′-ccggcatcgtagatcagc-3’	29
LAT4	SLC42A2	124935	NM_001284498.1NM_152346.2	F-5′-acaagtatggcccgaggaa-3’R-5′-gcaatcagcaagcaggaaa-3’	3

SLC, solute carrier; F, forward; R, reverse; 4F2HC, type-II membrane glycoprotein heavy chain.

**Table 2:** Information on metabolic genes, primers and probes.

Metabolic Genes	Gene ID	Genebank accession no.	Primers	Roche universal probe library no.
Branched chain aminotransferase 1mitochondrial ***(BCATm)***	587	NM_001190.2	F-5′-aaaatgggcctgagctgat-3’R-5′-gtgggctctgattccgtact-3’	17
Branched chain aminotransferase 2cytosolic ***(BCATc)***	586	NM_005504.5	F-5′-gatgtttggctctggtacagc-3’R-5′-ggaccattctccatagttggaa-3’	61
Glutaminase ***(GLS1)*** kidney isoform	2744	NM_014905.3	F-5′-tgcagagggtcatgttgaag-3’F-5′-catccatgggagtgttattcc-3’	11
Glutamine synthetase ***(GLUL)***	2752	NM_002065.4NM_001033044.1NM_001033056.1	F-5′-ccataccaacttcagcacca-3’R-5′-caatggcctcctcgatgta-3’	52
Glutamate dehydrogenase ***(GLUD)*** mitochondrial	2746	NM_005271.2NM_012084.3	F-5′-cactctggcttggcatacac-3’R-5′-tcaggtccaatcccaggtta-3’	76
Alanine aminotransferase 1 ***(GPT1)***	2875	NM_005309.2	F-5′-catagtgcagcgagccttg-3’R-5′-ggatgacctcggtgaaagg-3’	15
Alanine aminotransferase 2 ***(GPT2)***	84706	NM_133443.1	F-5′-ggatcttcattcctgccaaa-3’R-5′-acatgtctggagccatttga-3’	75
Aspartate aminotransferase 1 ***(GOT1***) cytosolic	2805	NM_002079.1	F-5′-caactgggattgacccaact-3’R-5′-ggaacagaaaccggtgctt-3’	38
Aspartate aminotransferase 2 ***(GOT2)*** mitochondrial	2806	NM_002080.2	F-5′-ccattctgaacaccccagat-3’R-5′-ggtcagccatgactttcactt-3’	46

F, forward; R, reverse

#### Probe and primer design

Oligonucleotide probes and primers were designed using the Roche ProbeFinder version 2.45 for human. Probes were supplied by Roche from the human universal probe library and primers were synthesised by Eurogentec (Seraing, Belgium). Control genes were selected using the geNormTM human Housekeeping Gene Selection Kit (Primer Design Limited, Southampton UK).

### Statistics

Summary data are presented as mean (SD) or median (inter-quartile range) depending on whether the data were normally distributed or not. Maternal variables that were not normally distributed were transformed logarithmically. Placental mRNA data were transformed to normality using a Fisher-Yates transformation [28], which converts the data to z-scores. Data were then adjusted for fetal sex, as gene expression was higher in placentas from male fetuses than in placentas from female fetuses [29]. Fetal variables were adjusted for sex [25], and neonatal measures were adjusted for sex and gestational age.

Relationships between placental gene expression levels and maternal, fetal or neonatal variables were analysed by linear regression and reported as correlation coefficients. Analysis of gene expression levels between different categories of maternal lifestyle were tested by one-way ANOVA. A significant difference or relationship was accepted at p < 0.05. To investigate whether there were sex differences in the relationship between mRNA expression and the maternal, fetal and infant variables, sex was included in regression analyses as appropriate and where a statistically significant interaction was found, data were analysed separately by sex. Data were analysed using Stata version 13.0 (Statacorp, Texas, USA).

## Results

### Characterisation of the subjects from the SWS cohort

The mean age (SD) of the 102 mothers at the birth of their child was 30.9 (3.9) years; the mean gestational age (SD) was 39.8 (1.3) weeks. The mean (SD) placental/fetal weight ratio was 0.13 (0.02). 53 of the infants were male and 49 were female. Alanine aminotransferase 1, liver isoform of glutaminase, *EAAT1*, *EAAT4* and *EAAT5* mRNA expression was not detected in human placenta.

### Maternal smoking

**Fig 1:**
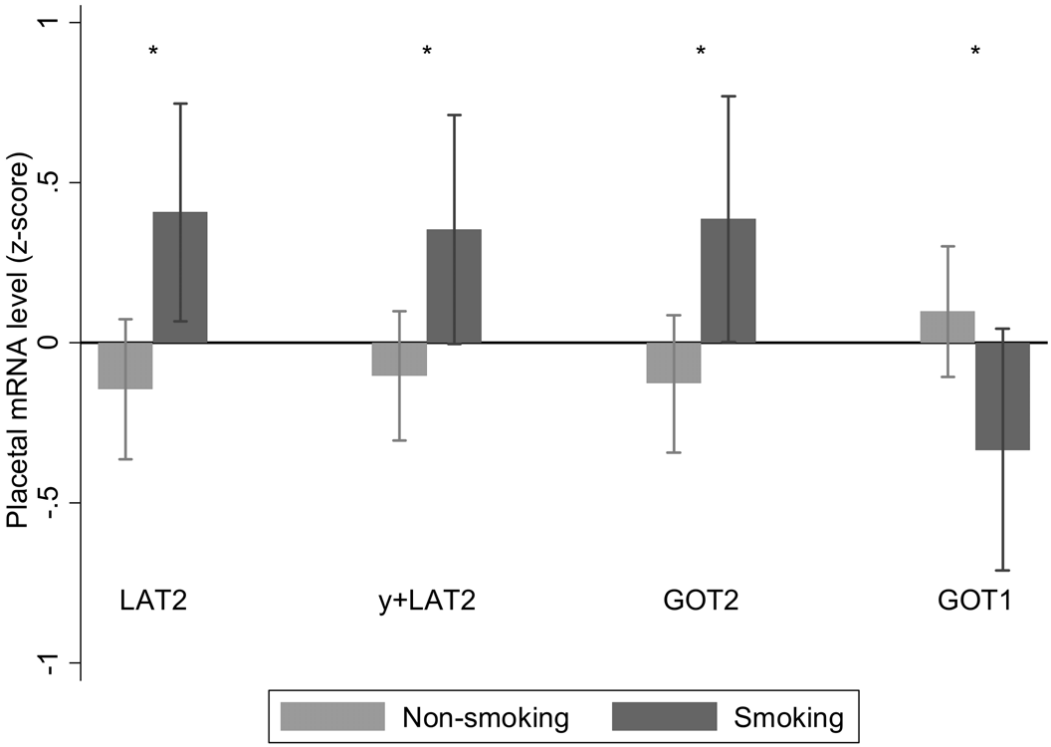
Associations between maternal pre-pregnancy smoking and placental relative mRNA expression levels. Maternal pre-pregnancy smoking was associated with increased *LAT2*, *y+LAT2* and aspartate aminotransferase 2 (*GOT2)* and decreased aspartate aminotransferase 1 (*GOT1)* placental relative mRNA expression; n = 76 for not smoking, n = 26 for smoking. Data are mean ± SD, * p < 0.05.

Pre-pregnancy maternal smoking was reported in 26 out of 102 women and was associated with increased placental *LAT2*, *y* ^*+*^ *LAT2* and aspartate aminotransferase 2 mRNA expression and decreased aspartate aminotransferase 1 mRNA expression (Fig 1A). There was an interaction between pre-pregnancy smoking and sex for *LAT3* and *y* ^*+*^ *LAT1* mRNA expression. Pre-pregnancy smoking was associated with higher *y* ^*+*^ *LAT1* mRNA expression in placentas of female (non-smoking -0.12 (0.89) n = 37; smokers 0.85 (0.74) n = 12; p = 0.001) but not male births (non-smoking -0.12 (0.94) n = 39; smokers -0.07 (1.1) n = 14; p = 0.86). Pre-pregnancy smoking was associated with higher *LAT3* mRNA expression in placentas of female (non-smoking 0.08 (0.87) n = 37; smokers 0.91 (0.86) n = 12; p = 0.006) but not male births (non-smoking -0.26 (0.93) n = 39; smokers -0.26 (0.96) n = 14; p = 0.98).

Smoking during pregnancy was reported in 14 out of 95 women. There was an interaction between smoking and sex for *y* ^*+*^ *LAT2* mRNA expression. In-pregnancy smoking was associated with higher *y* ^*+*^ *LAT2* mRNA expression in placentas of female (non-smoking 0.2 (0.9) n = 37; smokers 1.2 (0.9) n = 6; p = 0.01) but not male births (non-smoking -0.3 (0.9) n = 44; smokers -0.4 (1.0) n = 8; p = 0.70).

### Maternal exercise

**Fig 2:**
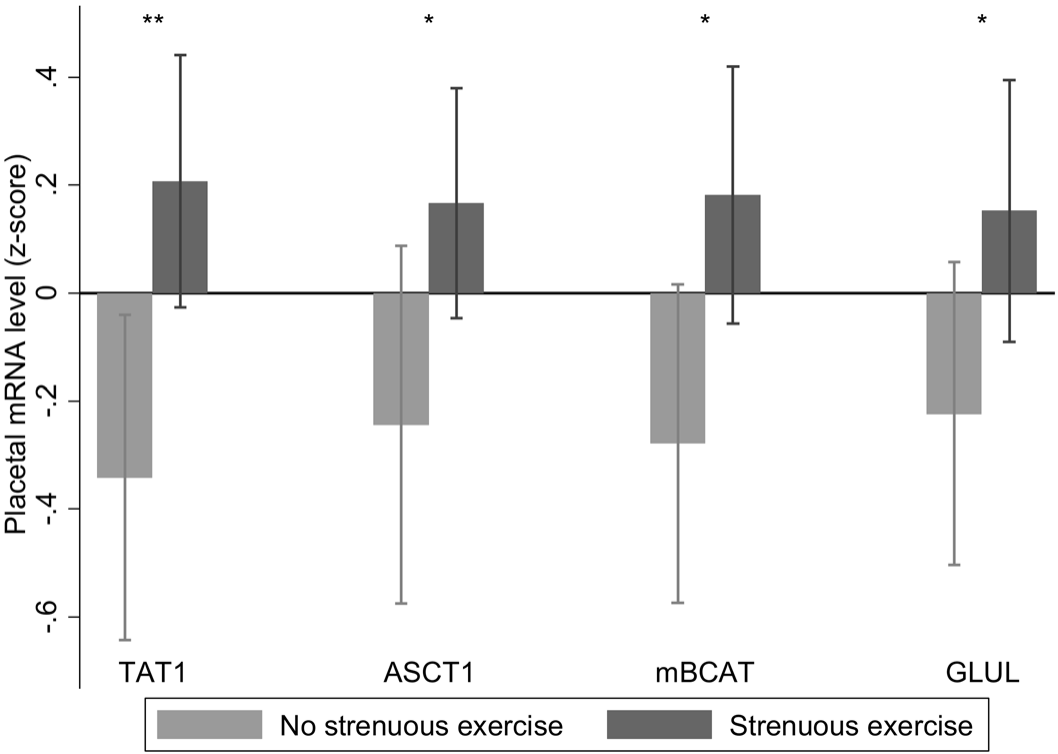
Maternal strenuous exercise was associated with increased placental *TAT1*, *ASCT1*, mitochondrial branched chain amino transferase (*BCATm)* and glutamine synthetase (*GLUL*) relative mRNA expression; n = 39 for no strenuous exercise, n = 63 for strenuous exercise. Data are mean ± SD, * p < 0.05, ** p < 0.001.

**Fig 3:**
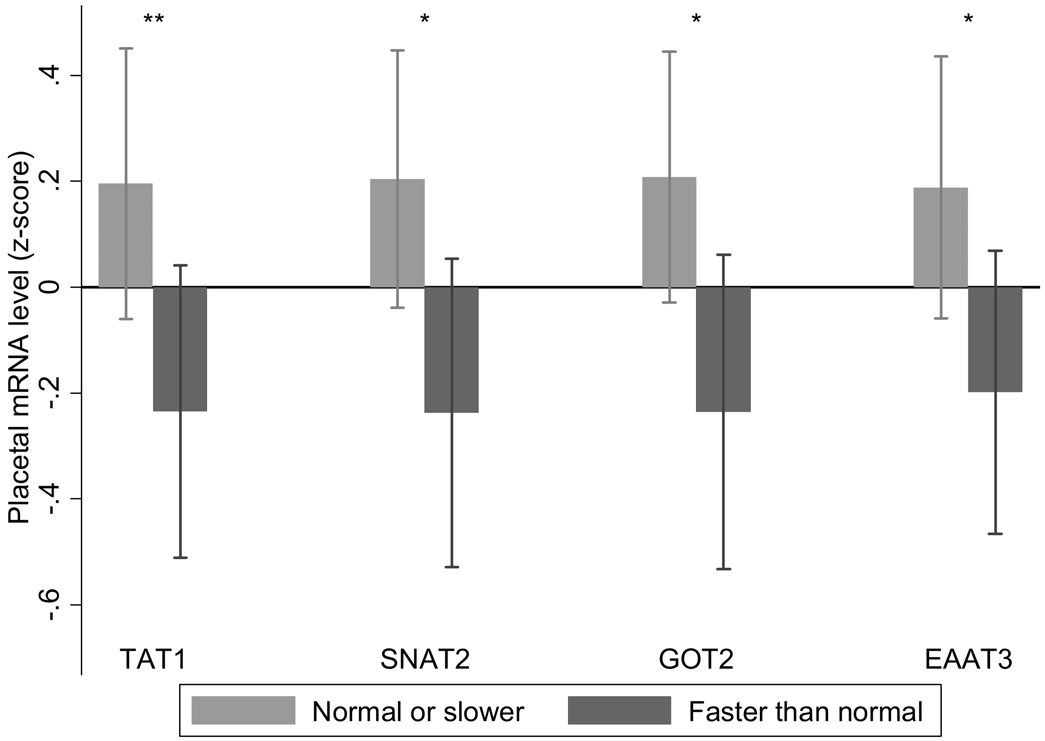
Faster walking speed was associated with reduced placental *TAT1*, *SNAT2*, aspartate aminotransferase 2 (*GOT2*) and *EAAT3* relative mRNA expression; n = 47 faster than normal walking, n = 55 for normal or slower walking speed. Data are mean ± SD, *p < 0.05, **p < 0.01.

At recruitment 60 out of 102 women answered ‘yes’ to participating in strenuous exercise, which was associated with increased *TAT1*, *ASCT1*, mitochondrial branched chain aminotransferase and glutamine synthetase mRNA expression (Fig 2). There was an interaction between strenuous exercise and sex of the fetus for glutaminase (kidney isoform) mRNA expression. This was higher with strenuous exercise in placentas of male (no strenuous exercise -0.6 (0.7) n = 23; strenuous exercise 0.2 (1.0) n = 30; p = 0.001) but not female births (no strenuous exercise 0.2 (0.8) n = 16; strenuous exercise 0.2 (1.0) n = 33; p = 0.95). Faster than normal walking speed was reported in 47 out of 102 women and was associated with decreased placental *TAT1*, *SNAT2*, aspartate aminotransferase 2 and *EAAT3* mRNA expression (Fig 3).

### Maternal diet and weight gain during pregnancy

Maternal dietary prudence before pregnancy was positively related to placental glutamine synthetase mRNA levels (r = 0.20, p = 0.05, n = 102) and at 11 weeks’ gestation (n = 79) was negatively related to placental *LAT4* (r = -0.24, p = 0.05) and aspartate aminotransferase 2 (r = -0.28, p = 0.01) mRNA levels.

Maternal high energy diet was associated negatively with placental *LAT4* mRNA levels at 11 weeks’ gestation (r = -0.27, p = 0.02, n = 79). There were interactions with sex and high energy diet for aspartate aminotransferase 1 and *EAAT2*. Aspartate aminotransferase 1 mRNA levels were positively associated with pre-pregnancy high energy diet in placentas of female (r = 0.29, p = 0.04, n = 49) but not male (r = -0.14, p = 0.31, n = 53) births. *EAAT2* mRNA levels were positively associated with pre-pregnancy high energy diet in placentas of male (r = 0.27, p = 0.05, n = 53) but not female (r = -0.15, p = 0.31, n = 49) births.

Maternal weight gain between conception and 34 weeks’ gestation (n = 87) was positively associated with placental *LAT3* (r = 0.25, p = 0.02) and *y* ^*+*^ *LAT2* (r = 0.24, p = 0.02) mRNA expression.

### Maternal birth weight

Maternal birth weight was negatively associated with placental mitochondrial branched chain aminotransferase mRNA levels (r = -0.27, p = 0.01, n = 88). There was an interaction between maternal birth weight and offspring sex for *ASCT1* and *LAT2* mRNA levels. *ASCT1* mRNA levels were positively related to maternal birth weight in placentas of female (r = 0.35, p = 0.02, n = 44) but not male (r = -0.28, p = 0.07, n = 44) births. *LAT2* mRNA levels were negatively related to maternal birth weight in placentas of male (r = -0.45, p = 0.002, n = 44) but not female (r = 0.17, p = 0.28, n = 43) births.

### Maternal body composition

Placental *ASC1* mRNA levels associated negatively with maternal pre-pregnancy BMI, sum of skinfold measures, fat mass and calf circumference as well as pre-pregnancy, 11 weeks’ gestation and 34 weeks’ gestation mid-upper arm circumference and arm muscle area (Table 3). Pre-pregnancy arm muscle area related to *SNAT1* mRNA expression (r = -0.24, p = 0.01, n = 101), and arm muscle area at 34 weeks’ gestation related to *SNAT4* mRNA expression (r = -0.21, p = 0.04, n = 95). Placental y^*+*^ *LAT2* mRNA levels were associated negatively with maternal pre-pregnancy BMI (r = -0.19, p = 0.05, n = 101) and fat mass (r = -0.20, p = 0.05, n = 101). Maternal pre-pregnancy calf circumference was negatively associated with placental mRNA levels (n = 100) for *LAT3* (r = -0.23, p = 0.02) and positively for *LAT4* (r = 0.21, p = 0.04). Maternal height (n = 101) was positively associated with placental aspartate aminotransferase 1 mRNA levels (r = 0.24, p = 0.01) and negatively associated with placental mRNA levels of cytosolic branched chain aminotransferase (r = -20, p = 0.05) and *SNAT1* (r = -29, p = 0.004). There was an interaction between maternal height and sex for mitochondrial branched chain aminotransferase mRNA levels. This gene was negatively associated with maternal height in placentas of male (r = -0.37, p = 0.01, n = 53) but not female (r = 0.07, p = 0.63, n = 48) births.

**Table 3:** Relationships between maternal body composition and *ASCT1* relative mRNA expression. Body mass index (BMI), sum of skinfolds (SSF), derived fat mass, calf circumference (circ), mid-arm upper circumference (MUAC), and arm muscle area (AMA). Pre = before pregnancy, 11-wk and 34-wk = gestation in weeks.

Maternal body composition (all log transformed)	*ASCT1 mRNA expression*		
	r	p	n
Pre BMI	**-0.26**	**0.01**	**101**
Pre SSF	**-0.20**	**0.04**	**100**
Pre fat mass(kg)	**-0.27**	**0.01**	**101**
Pre calf circ	**-0.34**	**0.00**	**100**
Pre MUAC	**-0.25**	**0.01**	**101**
11-wk MUAC	**-0.23**	**0.04**	**76**
34-wk MUAC	**-0.26**	**0.01**	**95**
Pre AMA	**-0.21**	**0.04**	**101**
11-wk AMA	**-0.23**	**0.05**	**76**
34-wk AMA	**-0.21**	**0.04**	**95**

### Parity

**Fig 4:**
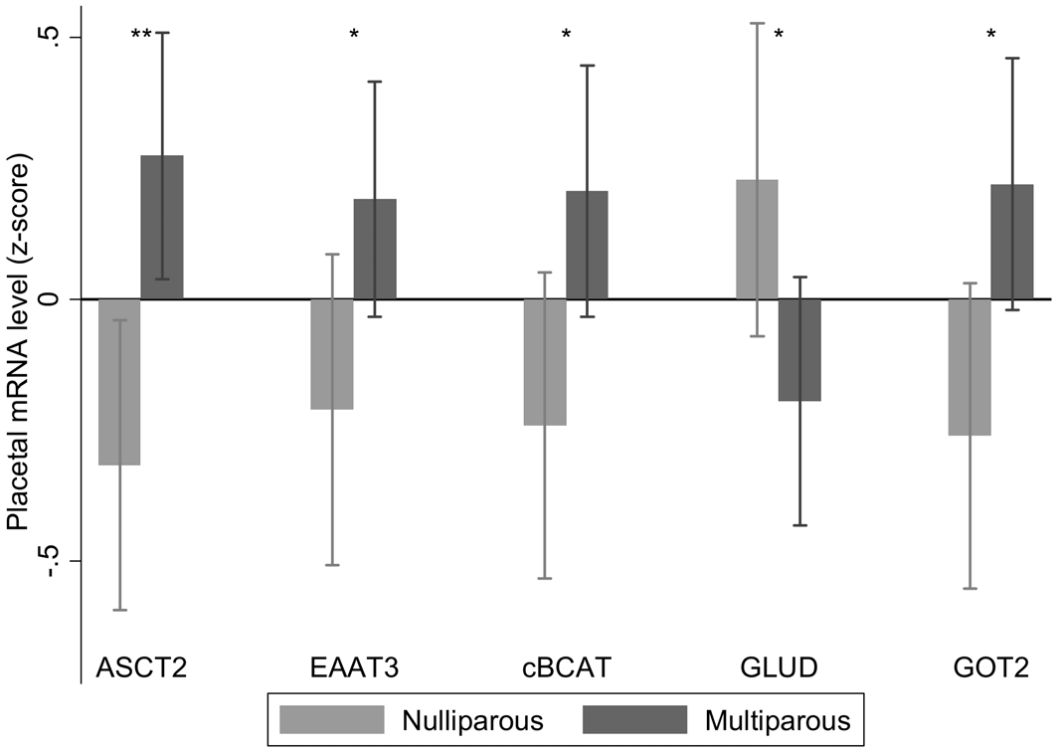
Placentas from multiparous woman had increased placental relative mRNA expression of *ASCT2*, *EAAT3*, cytosolic branched chain amino transferase (*BCATc)* and aspartate aminotransferase 2 (*GOT2)* and decreased mitochondrial glutamate dehydrogenase (*GLUD)*, n = 46 for nulliparous, n = 56 for multiparous. Data are mean ± SD, *p < 0.05, **p < 0.01.

56 women were multiparous (MP, 2 or more births) compared with 46 primiparous (PP, 1 birth) women. Multiparous women had increased *ASCT2*, aspartate aminotransferase 2, cytosolic branched chain aminotransferase and *EAAT3* and decreased mitochondrial glutamate dehydrogenase placental mRNA expression (Fig 4). There were interactions between parity and sex for alanine aminotransferase 2 and *LAT2* mRNA expression. Multiparous women had lower alanine aminotransferase 2 mRNA levels in placentas of male (PP, 0.42 (0.76), n = 23, MP, -0.32 (1.1), n = 30; p = 0.01), but not female births (PP, -0.02 (1.0); n = 23, MP, 0.02 (0.85), n = 26; p = 0.90). Increased parity was associated with higher *LAT2* mRNA levels in placentas of male (PP, -0.29 (1.0), n = 23, MP, 0.21 (0.79), n = 30; p = 0.05), but not female births (PP, 0.17 (0.87), n = 23; MP, -0.17 (1.1), n = 25; p = 0.24).

### Maternal social class and educational attainment

Maternal social class was recorded for 101 women and was associated with placental *4f2hc* mRNA levels (I/II -0.27 (0.87), n = 47; IIIN/M 0.23 (1.0), n = 43; IV/V 0.29 (0.66), n = 11; p = 0.03). There was an interaction between sex and social class for *LAT4* mRNA expression. In placentas for female births, *LAT4* mRNA levels were lower in placentas from women of social class I/II (p = 0.04). Maternal educational attainment was not related to the expression of any of the genes reported here.

### Fetal growth

Fetal head circumference at 11-weeks’ gestation was negatively associated with glutamine synthetase mRNA expression (r = -0.28, p = 0.05, n = 48). Head circumference at 19-weeks’ gestation was positively associated with placental *y* ^*+*^ *LAT2* mRNA expression (r = 0.22, p = 0.03, n = 94). Head circumference growth from 19 to 34 weeks’ gestation was negatively associated with *LAT2* (r = -0.24, p = 0.02, n = 93) and positively with *TAT1* (r = 0.27, p = 0.01, n = 94) mRNA expression. Abdominal circumference growth from 19 to 34 weeks’ gestation was positively associated with glutamine synthetase (r = 0.28, p = 0.01) and *TAT1* (r = 0.23, p = 0.03) mRNA expression (n = 94). Femur length growth from 19 to 34 weeks’ gestation was negatively associated with *SNAT2* mRNA expression (r = -0.21, p = 0.04, n = 94).

### Birth parameters

Placental *LAT2* mRNA expression (n = 100) was negatively associated with placental weight (r = -0.22, p = 0.03). Placental mRNA expression of alanine aminotransferase (n = 102) was negatively associated with birth weight (r = -0.20, p = 0.05), neonatal abdominal circumference (r = -0.25, p = 0.01), neonatal subscapular skinfold thickness (r = -0.33, p = 0.001), neonatal fat mass (r = -0.20, p = 0.04) and placental weight (r = -0.24, p = 0.02, n = 101). Subscapular skinfold thickness was also related to *LAT1* mRNA expression (r = -0.29, p = 0.003, n = 102). Neonatal head circumference was related to *y* ^*+*^ *LAT1* mRNA expression (r = 0.21, p = 0.03, n = 102).

## Discussion

This study identified a number of maternal factors associated with the expression of multiple genes in the placenta. As many of the factors are potentially modifiable, they may provide potential targets for improving placental function via lifestyle interventions. They may also help elucidate the underlying signalling pathways that modify placental function and fetal growth. Understanding the relationship between mother, placenta and fetus is important as fetal growth influences several aspects of offspring health, including the risk of developing chronic diseases in adulthood. This study has the advantage of using a well characterised population representative of the general population. However, the exploratory nature of this study, small sample size and the possibility of chance findings due to multiple testing need to be acknowledged. The observational nature of the study and co-linearity among both predictors and outcomes, meant testing for multiple comparisons was felt to be inappropriate [30]. In addition, some of the factors within the categorical data are generated from questionnaire data based on the mothers perceived or observed level of the particular factor rather than a measurable value, for example perceived walking speed. This may mean imprecise values in some cases and be a potential limitation to the study. Nevertheless, the patterns of observations are indicative of a role for maternal factors in the regulation of placental amino acid transporter and metabolic gene expression. If these associations are replicated in future studies they will provide modifiable targets for inclusion in intervention programs.

### Maternal smoking

Smoking during pregnancy may lead to reduced birth weight, infant morbidity and mortality, and increased risk of disease in later life [31]. Maternal smoking influences placental structure, transporter and enzyme activity [32] which may underlie the effects on fetal growth. We know that maternal smoking can alter the methylation of placental and embryonic genes which suggests a mechanism by which smoking influences gene expression[33]. In placentas of woman who smoke, cytochrome P450 1A1, which detoxifies compounds in tobacco smoke, has reduced DNA methylation within its promotor and increased gene expression [34]. This is consistent with the current study showing that the expression of five placental amino acid transport genes is altered by pre-pregnancy maternal smoking. The changes could either be a pathological response or a placental adaptation to a less favourable maternal environment. Many women in our cohort stopped smoking in pregnancy, reducing our sample size, we also cannot be sure as to whether the woman accurately reported their smoking status. However, smoking in pregnancy was associated with changes in the gene expression of several amino acid transporters and metabolic enzymes.

### Maternal exercise

Pregnant woman are recommended to exercise moderately to reduce the risk of pregnancy complications including back pain, mood disorders and to control excessive pregnancy weight gain, which can have adverse effects on the mother and the offspring[35,36]. As the activity of the amino acid transporter system A was associated with maternal muscle mass [37], it was interesting to see that mothers undertaking strenuous exercise and those with a faster than normal walking speed had altered placental expression of multiple genes including system A. Strenuous exercise was associated with up-regulation of certain genes, and increased walking speed associated with a down-regulation of others. The fact that strenuous exercise and faster walking speed affected different genes in opposite directions is difficult to explain and suggests that these surrogate measures of maternal exercise/fitness may reflect different aspects of maternal physiology. The fact that these factors are based on the mother’s perceived level of exercise and walking speed also needs to be acknowledged. Further work is required using better measures of maternal fitness.

### Maternal diet and body composition

Multiple measures of maternal body composition relating to muscle and fat mass were associated with *ASCT1* and *y* ^*+*^ *LAT2* mRNA levels. This suggests that these genes may be particularly responsive to signals indicative of maternal nutrient reserves [38,39]. These signals could be hormonal, such as glucocorticoids and thyroid hormones, which are known to regulate glutamine metabolism, or metabolic signals such as the levels of specific nutrients [40]. For instance, arm muscle area correlates with plasma amino acid composition in some populations [41]. Maternal fat and lean mass may determine the mother’s capacity to support the pregnancy, especially if food becomes scarce. *ASCT1* is known to be activated by nutrient deprivation [42] and the amino acid deprivation/integrated stress response pathway [43] as well as glutamate levels [44]. Maternal muscle measures also related to *LAT3* expression again indicating a response to maternal nutrient reserves. LAT3 expression is increased during starvation [45] suggesting a role in the metabolic cycle of branched-chain amino acids (BCAAs) consistent with the increased BCAA levels seen in the circulation during starvation [46]. LAT4 also transfers BCAAs from mother to fetus, but the positive relationship with maternal muscle levels and relationships with maternal diet indicate a differential regulation [47]. These findings may indicate the importance of the maternal diet before pregnancy in creating the environment to support pregnancy.

### Parity

Nulliparous women have higher rates of low birth weight offspring and/or placental weight [48,49]. It is thought that there is uterine adaptation during the first pregnancy resulting in greater maternal constraint which allows the mother to support later pregnancies better. The mechanisms underlying these observations are unknown. However, we observed changes in placental gene expression in the placentas of multiparous mothers which may reflect these changes in uterine environment and provide clues to their nature. Uterine changes during the first pregnancy may improve blood supply so the changes in placental gene expression may reflect changes in nutrient availability. Certain genes were also associated (in a sex specific manner) with both parity and placental weight, which is consistent with the reported association between parity and placental weight [48].

### Offspring sex

In placentas from female births, maternal smoking was associated with higher *LAT3* mRNA expression both before and during pregnancy. Placental gene expression is known to be fetal sex-specific [50] with placentas from female births being more sensitive to environmental changes such as glucocorticoid levels [51]. Under-nutrition during pregnancy also alters placental size and the placental programming of hypertension differently in males and females [11,52]. The placentas of female births respond to changes in the maternal environment whereas those for males appear to cope with these changes, making compensations that may put them at increased risk of disease in later life.

### Fetal placental relationships

While there were relatively few relationships between placental gene expression and fetal growth, an important association was seen between alanine aminotransferase 2 and several markers of fetal growth and body composition. Alanine aminotransferase 2 is an important enzyme in intermediary metabolism generating pyruvate and glutamate or alanine and α-ketogluterate and serum levels of this enzyme are positively associated with markers of metabolic syndrome [53]. Although measured at the mRNA level, the placental expression pattern of this enzyme may be a marker for particular fetal growth trajectories and subsequent risk of adulthood disease. As enzymes such as alanine aminotransferase are subject to post-translation modifications and allosteric regulation further studies should investigate associations between protein levels and these markers of fetal growth. The limited number of changes in placental gene expression in relation to fetal growth parameters may indicate that the placenta is able to adapt and normalise fetal growth in response to suboptimal conditions. However, while changes in the mRNA expression of placental genes reflect sensing mechanisms, changes in individual gene expression levels or even protein expression levels may not necessarily reflect placental function [54]. Particularly in the case of transporters, while up or down regulation may affect the activity of the transporter, gene expression or even activity cannot be assumed to correlate with increased or decreased placental transfer as a whole [55].

### Conclusion

In conclusion, this study demonstrates relationships between potentially modifiable maternal factors and placental gene expression which suggest that these maternal factors could influence placental function. These associations provide multiple avenues for more targeted investigations in the future. The relationships with maternal smoking and exercise are particularly interesting as these lifestyle factors are amenable to modification.

## Supporting Information

S1 File Relative mRNA levels and their relationships with maternal factors. Relationship between placental mRNA levels (adjusted for sex) at birth and maternal lifestyle (Table A). Relationship between placental mRNA levels (adjusted for sex) at birth and maternal diet and weight gain during pregnancy (Table B). Relationship between placental mRNA levels at birth (adjusted for sex) and maternal walking speed (Table C). Normalized placental amino acid transporter mRNA levels (Fisher-Yates transformed; Table D).Normalized placental amino acid metabolic enzyme mRNA levels (Fisher-Yates transformed; Table E).
(DOC)
