# Supplementary material for: Maternal Factors Are Associated with the Expression of Placental Genes Involved in Amino Acid Metabolism and Transport
Source: PLoS One. 2015 Dec 14;10(12):e0143653. doi: 10.1371/journal.pone.0143653 (PMC4682815; doi:10.1371/journal.pone.0143653)
Supplement: S1 File — Relationship between placental mRNA levels (adjusted for sex) at birth and maternal lifestyle (Table A). Relationship between placental mRNA levels (adjusted for sex) at birth and maternal diet and weight gain during pregnancy (Table B). Relationship between placental mRNA levels at birth (adjusted for sex) and maternal walking speed (Table C). Normalized placental amino acid transporter mRNA levels (Fisher-Yates transformed; Table D).Normalized placental amino acid metabolic enzyme mRNA levels (Fisher-Yates transformed; Table E). (DOC) [file pone.0143653.s001.doc]

Table A (i). Relationship between placental mRNA levels at birth (adjusted for sex) and maternal lifestyle

|  | TAT 1 | | LAT 3 | | LAT 4 | | SNAT 1 | | SNAT 2 | | SNAT 4 | |
| --- | --- | --- | --- | --- | --- | --- | --- | --- | --- | --- | --- | --- |
|  | Mean (SD) | P | Mean (SD) | P | Mean (SD) | P | Mean (SD) | P | Mean (SD) | P | Mean (SD) | P |
| Parity |  |  |  |  |  |  |  |  |  |  |  |  |
| 0 | -0.11(1.00) |  | 0.06(1.00) |  | 0.08(1.03) |  | -0.19(0.94) |  | 0.09(1.03) |  | 0.04(0.88) |  |
| 1+ | 0.09(0.93) | 0.29 | -0.03(0.87) | 0.66 | -0.08(0.89) | 0.41 | 0.15(0.97) | 0.08 | -0.07(0.91) | 0.42 | -0.01(0.96) | 0.76 |
| Pre pregnancy smoking status |  |  |  |  |  |  |  |  |  |  |  |  |
| No | -0.04(0.98) |  | -0.09(0.90) |  | 0.03(1.01) |  | -0.10(0.95) |  | -0.11(0.95) |  | 0.07(0.96) |  |
| Yes | 0.10(0.92) | 0.53 | 0.30(0.95) | 0.06 | -0.11(0.76) | 0.51 | 0.30(0.97) | 0.07 | 0.32(0.94) | 0.05 | -0.16(0.79) | 0.29 |
| In pregnancy smoking status |  |  |  |  |  |  |  |  |  |  |  |  |
| No | -0.04(0.95) |  | -0.07(0.89) |  | 0.06(1.00) |  | -0.01(0.93) |  | -0.04(0.97) |  | -0.02(0.89) |  |
| Yes | -0.05(0.90) | 0.97 | 0.36(0.86) | 0.10 | -0.25(0.71) | 0.26 | 0.37(1.01) | 0.16 | 0.15(1.07) | 0.52 | -0.05(0.96) | 0.89 |
| Strenuous exercise |  |  |  |  |  |  |  |  |  |  |  |  |
| No | -0.34(0.93) |  | -0.17(0.85) |  | -0.14(0.95) |  | -0.04(0.94) |  | -0.23(0.98) |  | -0.04(0.94) |  |
| Yes | 0.21(0.93) | <0.001 | 0.12(0.96) | 0.13 | 0.07(0.95) | 0.28 | 0.02(0.98) | 0.76 | 0.14(0.93) | 0.06 | 0.04(0.92) | 0.67 |

Table A (ii). Relationship between placental mRNA levels (adjusted for sex) at birth and maternal lifestyle

|  | LAT 1 | | LAT 2 | | ASCT 1 | | ASCT 2 | | Y+LAT 1 | |
| --- | --- | --- | --- | --- | --- | --- | --- | --- | --- | --- |
|  | Mean (SD) | P | Mean (SD) | P | Mean (SD) | P | Mean (SD) | P | Mean (SD) | P |
| Parity |  |  |  |  |  |  |  |  |  |  |
| 0 | 0.01 (0.96) |  | -0.06 (0.97) |  | 0.06 (0.93) |  | -0.32 (0.93) |  | -0.15 (0.91) |  |
| 1+ | -0.03 (0.93) | 0.830 | 0.04 (0.95) | 0.607 | -0.04 (0.95) | 0.594 | 0.27 (0.88) | 0.001 | 0.13 (0.98) | 0.143 |
| Pre pregnancy smoking status |  |  |  |  |  |  |  |  |  |  |
| No | 0.01 (0.97) |  | -0.14 (0.96) |  | 0.01 (0.91) |  | -0.08 (0.94) |  | -0.12 (0.92) |  |
| Yes | -0.07 (0.88) | 0.699 | 0.41 (0.82) | 0.011 | 0.02 (1.03) | 0.965 | 0.27 (0.93) | 0.098 | 0.36 (1.00) | 0.027 |
| In pregnancy smoking status |  |  |  |  |  |  |  |  |  |  |
| No | 0.05 (0.97) |  | -0.03 (1.02) |  | 0.01 (0.91) |  | -0.07 (0.90) |  | -0.05 (0.93) |  |
| Yes | -0.23 (0.90) | 0.317 | 0.30 (0.55) | 0.250 | 0.10 (1.05) | 0.743 | 0.17 (0.99) | 0.359 | 0.16 (0.92) | 0.434 |
| Strenuous exercise |  |  |  |  |  |  |  |  |  |  |
| No | -0.07 (0.86) |  | -0.22 (0.94) |  | -0.24 (1.02) |  | 0.01 (0.67) |  | -0.21 (0.86) |  |
| Yes | 0.03 (0.99) | 0.612 | 0.13 (0.94) | 0.078 | 0.17 (0.85) | 0.031 | 0.01 (1.09) | 0.976 | 0.14 (1.00) | 0.073 |

Table A (iii). Relationship between placental mRNA levels (adjusted for sex) at birth and maternal lifestyle

|  | Y+LAT 2 | | 4F2HC | | BCATc | | BCATm | | GLUD1/2 | | GLS1 | |
| --- | --- | --- | --- | --- | --- | --- | --- | --- | --- | --- | --- | --- |
|  | Mean (SD) | P | Mean (SD) | P | Mean (SD) | P | Mean (SD) | P | Mean (SD) | P | Mean (SD) | P |
| Parity |  |  |  |  |  |  |  |  |  |  |  |  |
| 0 | -0.09 (1.01) |  | -0.15 (0.90) |  | -0.24 (0.98) |  | 0.15 (0.91) |  | 0.23 (1.01) |  | -0.02 (0.95) |  |
| 1+ | 0.10 (0.81) | 0.298 | 0.11 (0.99) | 0.168 | 0.21 (0.90) | 0.018 | -0.12 (0.99) | 0.156 | -0.19 (0.89) | 0.027 | 0.03 (0.95) | 0.796 |
| Pre pregnancy smoking status |  |  |  |  |  |  |  |  |  |  |  |  |
| No | -0.10 (0.89) |  | -0.09 (0.90) |  | -0.06 (0.95) |  | -0.08 (0.94) |  | 0.07 (0.92) |  | 0.01 (0.94) |  |
| Yes | 0.35 (0.89) | 0.026 | 0.24 (1.08) | 0.128 | 0.19 (0.98) | 0.266 | 0.26 (0.98) | 0.121 | -0.23 (1.06) | 0.159 | 0.00 (0.99) | 0.971 |
| In pregnancy smoking status |  |  |  |  |  |  |  |  |  |  |  |  |
| No | -0.05 (0.90) |  | 0.01 (0.91) |  | -0.08 (0.92) |  | -0.06 (0.95) |  | 0.00 (0.97) |  | 0.05 (0.91) |  |
| Yes | 0.33 (1.06) | 0.162 | 0.23 (1.08) | 0.417 | 0.38 (0.85) | 0.090 | 0.32 (1.03) | 0.170 | -0.09 (0.98) | 0.739 | -0.36 (0.78) | 0.116 |
| Strenuous exercise |  |  |  |  |  |  |  |  |  |  |  |  |
| No | -0.09 (0.76) |  | -0.11 (1.01) |  | -0.05 (0.85) |  | -0.28 (0.91) |  | 0.07 (0.91) |  | -0.27 (0.77) |  |
| Yes | 0.08 (0.99) | 0.367 | 0.06 (0.92) | 0.388 | 0.04 (1.02) | 0.636 | 0.18 (0.95) | 0.017 | -0.05 (1.00) | 0.527 | 0.18 (1.01) | 0.019 |

Table A (iv). Relationship between placental mRNA levels (adjusted for sex) at birth and maternal lifestyle

|  | GLUl | | GPT2 | | GOT2 | | GOT1 | | EAAT2 | | EAAT3 | |
| --- | --- | --- | --- | --- | --- | --- | --- | --- | --- | --- | --- | --- |
|  | Mean (SD) | P | Mean (SD) | P | Mean (SD) | P | Mean (SD) | P | Mean (SD) | P | Mean (SD) | P |
| Parity |  |  |  |  |  |  |  |  |  |  |  |  |
| 0 | 0.13 (0.96) |  | 0.20 (0.91) |  | -0.26 (0.98) |  | 0.10 (0.86) |  | 0.02 (1.00) |  | -0.21 (1.00) |  |
| 1+ | -0.09 (0.92) | 0.226 | -0.16 (0.98) | 0.057 | 0.22 (0.90) | 0.011 | -0.10 (0.96) | 0.278 | -0.00 (0.90) | 0.896 | 0.19 (0.84) | 0.030 |
| Pre pregnancy smoking status |  |  |  |  |  |  |  |  |  |  |  |  |
| No | 0.07 (0.88) |  | 0.02 (0.96) |  | -0.13 (0.94) |  | 0.10 (0.89) |  | -0.02 (0.96) |  | -0.07 (0.90) |  |
| Yes | -0.18 (1.09) | 0.246 | -0.06 (1.01) | 0.702 | 0.39 (0.95) | 0.018 | -0.33 (0.94) | 0.038 | 0.08 (0.90) | 0.657 | 0.23 (1.00) | 0.163 |
| In pregnancy smoking status |  |  |  |  |  |  |  |  |  |  |  |  |
| No | 0.05 (0.89) |  | -0.02 (0.95) |  | -0.05 (0.95) |  | 0.10 (0.84) |  | -0.01 (1.00) |  | -0.08 (0.93) |  |
| Yes | -0.32 (1.29) | 0.184 | -0.13 (1.05) | 0.704 | 0.36 (1.04) | 0.143 | -0.34 (1.00) | 0.087 | 0.34 (0.43) | 0.196 | 0.43 (0.92) | 0.060 |
| Strenuous exercise |  |  |  |  |  |  |  |  |  |  |  |  |
| No | -0.22 (0.86) |  | -0.16 (0.99) |  | 0.09 (0.87) |  | 0.03 (0.87) |  | 0.13 (0.98) |  | -0.17 (0.82) |  |
| Yes | 0.15 (0.96) | 0.049 | 0.10 (0.94) | 0.195 | -0.05 (1.02) | 0.453 | -0.04 (0.96) | 0.730 | -0.07 (0.92) | 0.312 | 0.12 (0.99) | 0.126 |

Table B. Relationship between placental mRNA levels (adjusted for sex) at birth and maternal diet and weight gain during pregnancy

|  | Pre pregnancy dietary prudence (n=102) | | Pre pregnancy high energy diet (n=102) | | 11 week gestation dietary prudence (n=79) | | 11 week gestation high energy diet (n=79) | | Weight gain  (n=87) | |
| --- | --- | --- | --- | --- | --- | --- | --- | --- | --- | --- |
|  | r | P | r | P | r | P | r | P | r | P |
| TAT 1 | -0.01 | 0.90 | -0.04 | 0.70 | -0.09 | 0.45 | -0.02 | 0.88 | 0.15 | 0.16 |
| LAT 3 | 0.08 | 0.43 | -0.06 | 0.56 | -0.08 | 0.48 | 0.02 | 0.85 | 0.25 | 0.02 |
| LAT 4 | -0.13 | 0.19 | -0.08 | 0.43 | -0.24 | 0.03 | -0.27 | 0.02 | -0.02 | 0.84 |
| SNAT 1 | -0.13 | 0.19 | -0.06 | 0.56 | -0.20 | 0.08 | -0.03 | 0.79 | 0.05 | 0.63 |
| SNAT 2 | 0.00 | 0.98 | -0.09 | 0.38 | -0.15 | 0.18 | -0.08 | 0.49 | 0.16 | 0.15 |
| SNAT 4 | -0.17 | 0.08 | -0.09 | 0.39 | -0.16 | 0.15 | -0.14 | 0.22 | 0.17 | 0.12 |
| LAT 1 | 0.04 | 0.67 | 0.03 | 0.75 | -0.02 | 0.83 | 0.00 | 0.97 | -0.03 | 0.775 |
| LAT 2 | -0.14 | 0.17 | 0.18 | 0.07 | -0.22 | 0.06 | 0.07 | 0.57 | 0.05 | 0.647 |
| ASCT 1 | 0.08 | 0.40 | 0.03 | 0.75 | 0.05 | 0.63 | 0.10 | 0.39 | 0.11 | 0.311 |
| ASCT 2 | 0.03 | 0.75 | -0.03 | 0.78 | -0.08 | 0.47 | 0.19 | 0.10 | 0.10 | 0.369 |
| Y+LAT 1 | 0.12 | 0.21 | -0.04 | 0.72 | -0.04 | 0.73 | 0.07 | 0.51 | 0.09 | 0.382 |
| Y+LAT 2 | -0.09 | 0.38 | -0.02 | 0.85 | -0.14 | 0.21 | 0.12 | 0.29 | 0.24 | 0.024 |
| 4F2HC | -0.13 | 0.20 | 0.06 | 0.58 | -0.09 | 0.44 | 0.02 | 0.84 | 0.11 | 0.312 |
| BCATc | -0.04 | 0.72 | 0.01 | 0.95 | -0.19 | 0.09 | 0.05 | 0.66 | 0.02 | 0.886 |
| BCATm | -0.02 | 0.86 | -0.07 | 0.46 | -0.10 | 0.36 | -0.03 | 0.78 | 0.16 | 0.142 |
| GLUD1/2 | 0.03 | 0.75 | 0.08 | 0.40 | 0.17 | 0.14 | -0.01 | 0.96 | -0.06 | 0.577 |
| GLS1 | 0.03 | 0.74 | -0.04 | 0.67 | -0.09 | 0.44 | -0.06 | 0.58 | 0.05 | 0.644 |
| GLUl | 0.21 | 0.03 | -0.10 | 0.33 | 0.12 | 0.31 | -0.10 | 0.40 | -0.12 | 0.279 |
| GPT2 | -0.03 | 0.75 | 0.02 | 0.82 | -0.02 | 0.84 | -0.11 | 0.35 | 0.00 | 0.993 |
| GOT2 | -0.11 | 0.28 | -0.13 | 0.21 | -0.28 | 0.01 | 0.03 | 0.81 | 0.07 | 0.504 |
| GOT1 | -0.02 | 0.84 | 0.11 | 0.29 | -0.08 | 0.50 | -0.13 | 0.26 | -0.02 | 0.885 |
| EAAT2 | -0.01 | 0.93 | 0.05 | 0.59 | -0.00 | 0.99 | 0.18 | 0.12 | 0.06 | 0.611 |
| EAAT3 | -0.13 | 0.21 | -0.10 | 0.32 | -0.16 | 0.16 | 0.03 | 0.82 | -0.02 | 0.835 |

Table C. Relationship between placental mRNA levels at birth (adjusted for sex) and maternal walking speed

|  | Normal speed or slower (n=55) | | Faster than normal (n=47) | |  |
| --- | --- | --- | --- | --- | --- |
|  | Mean | SD | Mean | SD | P |
| TAT 1 | 0.20 | 0.95 | -0.24 | 0.94 | 0.02 |
| LAT 3 | 0.16 | 0.96 | -0.17 | 0.86 | 0.07 |
| LAT 4 | 0.00 | 0.97 | -0.01 | 0.94 | 0.94 |
| SNAT 1 | 0.13 | 1.10 | -0.16 | 0.77 | 0.13 |
| SNAT 2 | 0.20 | 0.90 | -0.24 | 0.99 | 0.02 |
| SNAT 4 | 0.12 | 0.89 | -0.12 | 0.96 | 0.19 |
| LAT 1 | 0.04 | 0.97 | -0.06 | 0.91 | 0.607 |
| LAT 2 | 0.11 | 1.02 | -0.14 | 0.87 | 0.180 |
| ASCT 1 | -0.01 | 1.04 | 0.04 | 0.80 | 0.799 |
| ASCT 2 | 0.17 | 0.92 | -0.18 | 0.95 | 0.067 |
| Y+LAT 1 | 0.15 | 0.89 | -0.16 | 1.02 | 0.109 |
| Y+LAT 2 | 0.10 | 0.86 | -0.09 | 0.95 | 0.285 |
| 4F2HC | 0.10 | 1.00 | -0.12 | 0.89 | 0.251 |
| BCATc | 0.09 | 1.05 | -0.10 | 0.84 | 0.309 |
| BCATm | 0.15 | 0.98 | -0.17 | 0.91 | 0.096 |
| GLUD1/2 | -0.11 | 1.05 | 0.12 | 0.84 | 0.243 |
| GLS1 | 0.17 | 0.95 | -0.19 | 0.91 | 0.054 |
| GLUl | -0.01 | 0.95 | 0.03 | 0.94 | 0.868 |
| GPT2 | 0.08 | 1.02 | -0.09 | 0.90 | 0.398 |
| GOT2 | 0.21 | 0.88 | -0.24 | 1.01 | 0.019 |
| GOT1 | -0.06 | 0.86 | 0.05 | 0.99 | 0.543 |
| EAAT2 | -0.02 | 0.93 | 0.04 | 0.97 | 0.748 |
| EAAT3 | 0.19 | 0.92 | -0.20 | 0.91 | 0.036 |

Table D. Normalized placental amino acid transporter mRNA levels (Fisher-Yates transformed)

| **PLACENTA** | | **TAT1** | **LAT3** | **LAT4** | **SNAT1** | **SNAT2** | **SNAT4** | **LAT1** | **LAT2** | **ASCT1** | **ASCT2** | **y+LAT1** | **y+LAT2** | **4F2HC** | **EAAT2** | **EAAT3** |
| --- | --- | --- | --- | --- | --- | --- | --- | --- | --- | --- | --- | --- | --- | --- | --- | --- |
| 1 | -0.27 | | 0.79 | 0.87 | 1.23 | 0.99 | 0.7 | -0.35 | -1.2 | -0.07 | -0.41 | -0.78 | -0.96 | -1.8 | 0.85 | -1.4 |
| 2 | 0.59 | | 1.49 | 0.84 | 0.78 | 1.01 | 0.71 | -2.28 | 0.36 | 2 | -0.47 | -0.27 | -0.56 | -2.48 | 0.4 | -0.19 |
| 3 | 0.5 | | 1.34 | 1.13 | 0.92 | 1.01 | 0.28 | -0.63 | -1.5 | 1.1 | 0.56 | -0.62 | 1.07 | -0.84 | 0.98 | 0.8 |
| 4 | -0.14 | | 0.79 | 1.29 | 0.98 | 0.84 | 0.86 | 1.36 | -0.81 | -0.25 | 0.48 | -1.03 | -0.42 | -1.34 | -1.03 | 0.36 |
| 5 | -0.34 | | 0.87 | 0.76 | 1.31 | 1.51 | 0.8 | -0.58 | -0.94 | -1.48 | 0.14 | -0.58 | -1.35 | -1.15 | 0.68 | -1.12 |
| 6 | -0.87 | | 0.84 | 1.27 | 1.67 | 0.95 | 1.15 | -0.1 | -1.57 | -1.06 | -0.08 | -0.46 | -1.08 | -1.04 | 0.3 | -0.57 |
| 7 | 0.19 | | 1.5 | 1.35 | 1.53 | 0.92 | 0.47 | -0.12 | 0 | 0.15 | 0.53 | 0.22 | 0.02 | -1.28 | 0.58 | 0.21 |
| 8 | -0.49 | | 1.35 | 1.19 | 0.83 | 1.19 | 0.49 | -2.12 | 1.5 | 1 | -0.56 | 0.14 | -0.42 | -0.16 | -1.19 | -0.64 |
| 9 | -0.06 | | 0.99 | 0.79 | 0.7 | 0.87 | 0.29 | -0.73 | -0.69 | -0.69 | 0.41 | 0.4 | -0.36 | -1.12 | 0.2 | -0.38 |
| 10 | 0.63 | | 0.9 | 1.13 | 1.42 | 0.94 | 1.44 | -1.28 | -0.67 | -1.43 | 0.74 | -0.25 | 0.24 | -0.06 | -0.01 | -0.8 |
| 11 | 1.23 | | 1.95 | 0.97 | 0.68 | 1.45 | 2.16 | -1.45 | -1.61 | 1.83 | 1.87 | 1.95 | 1.02 | -1.92 | -0.6 | 1.1 |
| 12 | -0.36 | | 1.07 | 1.11 | 1.18 | 1.13 | 1.4 | -0.25 | -1.91 | -0.03 | -0.38 | -1.09 | -0.32 | -1.42 | 1.85 | 0.61 |
| 13 | -0.21 | | 1.24 | 1.46 | 1.09 | 1.05 | 0.55 | -1.57 | 1.14 | 0.55 | -0.81 | 1.68 | -0.91 | -0.97 | -1.14 | -0.8 |
| 14 | -0.04 | | 0.72 | 1.74 | 1.25 | 0.66 | 0.55 | 1.08 | -1.5 | 0.49 | -0.11 | -1.41 | -0.35 | 0.3 | 0.12 | -0.34 |
| 15 | 0.25 | | 1.07 | 1.33 | 1.05 | 1.09 | 0.71 | -0.85 | -0.03 | -1.18 | -0.35 | 1.77 | -0.3 | -0.64 | -0.61 | -0.68 |
| 16 | -0.11 | | 0.92 | 1.33 | 1 | 0.89 | 0.92 | -0.5 | 0.6 | 0.03 | -1.87 | -1.24 | -1.73 | -0.4 | -0.88 | -0.89 |
| 17 | 0.14 | | 1.02 | 1.05 | 1.36 | 1.23 | 1.45 | 0.35 | -0.17 | 0.4 | -0.31 | -0.72 | 0.1 | -0.43 | 0.76 | 0.84 |
| 18 | -0.17 | | 0.81 | 1.53 | 0.87 | 0.95 | 0.39 | -0.52 | 0.25 | -1.25 | -0.44 | -1.65 | -0.53 | -0.78 | -1.55 | -0.54 |
| 19 | 0.1 | | 0.78 | 1.66 | 1.47 | 1.19 | 0.82 | 1.14 | -0.72 | -0.02 | -0.6 | -0.94 | -0.76 | -0.94 | 0.82 | -1.64 |
| 20 | -0.16 | | 0.89 | 1.21 | 1.41 | 1.1 | 1.04 | -0.76 | 0.3 | 0.2 | -1.25 | -0.55 | -0.25 | 1.12 | -0.08 | -0.36 |
| 21 | -0.11 | | 0.93 | 1.1 | 1.15 | 1.11 | 0.81 | -0.3 | 0.2 | 0.18 | -0.19 | -1.46 | -0.05 | -0.11 | 1.04 | 0.29 |
| 22 | 0.05 | | 0.85 | 0.96 | 1.47 | 1.21 | 0.91 | -0.15 | -1.08 | -0.34 | 0.31 | -0.44 | 0.08 | -0.87 | -2.12 | -0.99 |
| 23 | -0.16 | | 0.71 | 1.11 | 1.02 | 0.89 | 0.4 | 0.39 | -0.27 | -1.52 | -0.06 | 0.22 | -1.02 | 0.55 | -1.08 | 0.67 |
| 24 | 0.52 | | 0.73 | 1.38 | 1.23 | 0.99 | 1.54 | -0.4 | 0.74 | -0.43 | 0.21 | -0.12 | 0.37 | 0.4 | 0.28 | -0.94 |
| 25 | -0.16 | | 0.71 | 1.49 | 1.15 | 1.09 | 0.55 | -1.11 | 1.26 | -0.31 | -0.27 | -1.95 | -0.33 | 0.52 | 0.01 | -1.32 |
| 26 | 0.73 | | 0.88 | 1.56 | 1.79 | 1.2 | 0.85 | -0.55 | -0.25 | 0.66 | 1.16 | 0.3 | 0.76 | 0.45 | 0.48 | -0.61 |
| 27 | 0.55 | | 1.01 | 1.43 | 1.86 | 1.28 | 0.73 | 0 | 0.39 | 0.13 | -1.16 | -1.08 | -1.15 | -0.43 | -1.68 | -0.03 |
| 28 | -0.29 | | 0.86 | 1.47 | 2.23 | 1.14 | 0.69 | 0.55 | -1.74 | -0.63 | -1.1 | -1.21 | -0.4 | 1.01 | -0.74 | -0.19 |
| 29 | -0.34 | | 0.64 | 1.14 | 1.48 | 1.29 | 0.47 | -0.05 | 0.35 | -0.23 | -0.36 | -1.6 | -0.13 | 0.75 | -1.27 | -0.49 |
| 30 | -0.06 | | 0.87 | 1.47 | 1.63 | 1.44 | 1.27 | 0.72 | -1.12 | -0.55 | 0.59 | -0.71 | 0.4 | -0.04 | -0.48 | -0.35 |
| 31 | -0.16 | | 0.59 | 1.86 | 1.03 | 0.99 | 0.6 | -1.27 | 0.07 | -1.65 | -0.74 | -1.78 | -0.02 | -0.67 | 0.88 | -1.81 |
| 32 | 0.27 | | 1.08 | 1.16 | 1.47 | 1.29 | 1.13 | -2.11 | 0.1 | 0.16 | -0.95 | -0.3 | -0.13 | 0.75 | -0.2 | 0.61 |
| 33 | -0.64 | | 0.66 | 1.48 | 1.06 | 0.93 | 0.52 | 0.23 | -1.04 | -1.9 | -0.72 | -0.87 | -0.78 | -1.35 | -0.98 | -0.17 |
| 34 | -0.55 | | 0.58 | 1.55 | 1.34 | 0.91 | 0.5 | 0.76 | -0.6 | -0.94 | -1.3 | -1.77 | -1.91 | -0.27 | -0.5 | -2.02 |
| 35 | 0.01 | | 1.04 | 1.48 | 1.25 | 1.35 | 1.3 | -0.13 | -0.84 | -0.73 | 0.06 | -0.38 | -0.81 | 0.22 | -1.85 | 0.07 |
| 36 | -0.04 | | 0.92 | 1.84 | 1.12 | 1.23 | 0.93 | 1.36 | -0.23 | -0.88 | -1.06 | -0.3 | -0.92 | 0.43 | 0.02 | -0.02 |
| 37 | -0.38 | | 1.01 | 1.81 | 1.8 | 1.39 | 0.69 | -0.45 | -0.12 | 0.95 | -2.09 | -0.75 | -2.67 | 1.24 | 0.25 | -0.78 |
| 38 | -0.55 | | 0.64 | 1.14 | 1.05 | 0.71 | 0.9 | -0.57 | -0.13 | -0.77 | -2.14 | -2.22 | -1.56 | -0.16 | -0.42 | -2.08 |
| 39 | 0.21 | | 1.46 | 1.81 | 1.51 | 1.46 | 1.9 | -0.05 | 0.31 | 0.33 | -1.57 | -0.17 | 0.05 | -0.01 | -0.45 | 0.27 |
| 40 | -0.07 | | 1.03 | 1.79 | 1.85 | 1.32 | 1.22 | -0.32 | -1.04 | 0.08 | -1.46 | 1.21 | -0.72 | 0.49 | -0.65 | 0.05 |
| 41 | -0.23 | | 0.8 | 1.68 | 1.54 | 1.44 | 1.06 | 1.7 | 0.25 | -0.68 | -0.9 | -0.81 | -1.13 | -0.01 | -0.52 | 0 |
| 42 | 0.69 | | 1.46 | 1.08 | 1.05 | 1.25 | 0.54 | -0.98 | -0.99 | 0.52 | 0.21 | 0.82 | 0.46 | 0.01 | -1.71 | 0.54 |
| 43 | -0.32 | | 1.26 | 1.3 | 1.05 | 1.29 | 0.68 | 0.25 | 0.17 | 0.71 | -0.99 | 0.2 | 0.15 | 0.58 | 0.47 | 0.54 |
| 44 | 0.38 | | 1.31 | 1.75 | 1.62 | 1.01 | 1.23 | 0.91 | 1.16 | -0.13 | -1.44 | 0.52 | -0.45 | 0.61 | -1.01 | -0.51 |
| 45 | -0.33 | | 0.95 | 1.93 | 1.3 | 0.92 | 1.52 | 0.57 | -0.7 | -1.07 | -0.02 | -1.53 | 1.08 | 0.38 | 1.68 | -1.55 |
| 46 | 0.39 | | 1.06 | 1.96 | 1.28 | 1.18 | 0.73 | 0.65 | 0.94 | 0.69 | -1.77 | -0.91 | -0.67 | 0.56 | -0.4 | -1.15 |
| 47 | -0.74 | | 0.81 | 1.47 | 1.66 | 1.39 | 1.59 | 0.18 | -1.41 | -0.65 | -0.53 | -0.05 | 0.64 | 0.16 | -0.11 | -0.27 |
| 48 | 0.39 | | 1.45 | 1.46 | 1.4 | 1.18 | 1.18 | -1.23 | -1.64 | 0.65 | -0.21 | 0.64 | 0.2 | -0.21 | 1.71 | 0.92 |
| 49 | 0.86 | | 1.02 | 1 | 1.31 | 1.17 | 1.16 | -1.98 | -1.16 | 0.76 | 1.5 | 1.01 | -0.08 | -0.06 | -0.39 | -0.47 |
| 50 | 0.64 | | 1.25 | 1.62 | 0.92 | 1.11 | 1.35 | -0.45 | -0.94 | 0.85 | -1.1 | 0.61 | 0.75 | -1.24 | 0.94 | 0.44 |
| 51 | -0.16 | | 1.05 | 1.36 | 1.8 | 0.99 | 0.67 | -0.23 | -0.32 | 0.3 | -0.01 | 1.88 | 0.62 | -0.48 | 0.06 | -1.24 |
| 52 | -0.16 | | 1.16 | 1.38 | 1.28 | 1.58 | 0.78 | 0.45 | 0.7 | -0.37 | 0.36 | -0.09 | 1.53 | 0.65 | 0.15 | -0.76 |
| 53 | 0.61 | | 1.17 | 1 | 0.76 | 1.11 | 0.94 | -0.79 | -1.44 | 1.34 | 0.33 | 0.32 | -0.39 | -0.23 | -1.35 | -0.21 |
| 54 | 0.15 | | 1.16 | 1.61 | 2.56 | 1.19 | 0.9 | 0.28 | 1.09 | 1.9 | -0.53 | 0.13 | -0.86 | 0.87 | -0.12 | 1.08 |
| 55 | -0.06 | | 1.1 | 1.36 | 1.04 | 0.82 | 0.7 | -0.52 | -0.31 | -0.58 | -0.69 | -0.15 | -0.15 | 0.35 | -1.15 | 0.51 |
| 56 | 0.33 | | 1.06 | 1.26 | 1.37 | 1.67 | 1.25 | -0.48 | 2.04 | 0.09 | 0.56 | -0.42 | -0.5 | 0.21 | 0.31 | 0.47 |
| 57 | -0.34 | | 1.13 | 1.37 | 1.58 | 1.47 | 0.58 | -0.28 | 2.49 | 1.18 | -1.17 | 0.49 | -0.03 | 0.11 | -0.79 | 0.64 |
| 58 | 0.45 | | 1.17 | 1.22 | 1.4 | 1.5 | 0.71 | 0.5 | 1.81 | 0.77 | 0.27 | 1.24 | 0.56 | 0.58 | -1.23 | 1.04 |
| 59 | 0.51 | | 1.34 | 1.54 | 2.06 | 1.46 | 1.03 | 0.69 | 0.54 | 0.94 | 1.17 | 0.91 | 1.48 | 1.21 | 1.41 | 1.82 |
| 60 | 0 | | 1.23 | 1.58 | 1.59 | 1.16 | 0.92 | 1.09 | 1.61 | 0.47 | 0.9 | 1.13 | 0.94 | 1.51 | -0.33 | 0.12 |
| 61 | -0.17 | | 1.09 | 1.57 | 1.78 | 1.26 | 0.88 | 0.42 | 0.82 | 0.2 | 1.62 | 1.31 | 1.03 | 1.92 | -0.04 | 0.64 |
| 62 | 0.41 | | 1.36 | 1.55 | 1.83 | 1.23 | 0.61 | 0.1 | 0.9 | 0.6 | 0.75 | 0.98 | 1 | 0.68 | 0.76 | 1.4 |
| 63 | 1.07 | | 1.8 | 1.37 | 3.07 | 1.46 | 1.14 | 1.19 | 1.64 | 1.06 | 0.24 | 0.86 | 0.05 | 1.91 | -0.68 | -0.83 |
| 64 | 0.45 | | 0.86 | 1.27 | 1.31 | 1 | 0.76 | 0 | 0.05 | -0.11 | -0.64 | 0.07 | 0 | -0.4 | -0.58 | -0.92 |
| 65 | -0.46 | | 1.2 | 1.26 | 1.27 | 1.23 | 0.76 | 0.98 | -0.18 | -1.12 | -0.56 | 0 | -1.43 | 0.11 | 0.23 | -1.17 |
| 66 | -0.06 | | 0.82 | 1.5 | 1.66 | 1 | 0.83 | 0.97 | 0.1 | -0.76 | 1.37 | 0.12 | -0.23 | 0.33 | 0.88 | -0.1 |
| 67 | 0.75 | | 1.19 | 1.53 | 1.44 | 0.99 | 0.91 | 0.31 | 0.42 | -0.62 | 0.51 | -0.25 | 0.86 | -1.08 | 1.51 | 0.95 |
| 68 | 0.29 | | 0.92 | 1.53 | 1.83 | 1.16 | 1.2 | -1.04 | -0.4 | -0.86 | 0.99 | 0.69 | -0.16 | -0.25 | 0.58 | 0.19 |
| 69 | 0.02 | | 0.74 | 1.78 | 1.27 | 1.16 | 0.64 | -1.36 | 0.08 | -2.31 | -0.43 | -1.05 | -0.86 | -0.72 | -0.45 | -1.23 |
| 70 | 0.02 | | 0.91 | 1.44 | 1.26 | 1.07 | 0.65 | 0.52 | 0.51 | -0.9 | 0.46 | 0.27 | 0.5 | 0.24 | -0.28 | 0.78 |
| 71 | -0.48 | | 0.95 | 1.31 | 2.09 | 1.04 | 1.19 | 0.05 | 0.28 | -2.1 | -0.11 | -0.9 | -0.64 | 0.62 | 0.18 | 0.46 |
| 72 | -0.38 | | 1.02 | 1.59 | 1.6 | 1 | 0.74 | 0.2 | 0.94 | 0.52 | 0.87 | 0.81 | -1.32 | 1 | -0.68 | 0.22 |
| 73 | 0.58 | | 1.39 | 1.38 | 1.65 | 1.42 | 1.05 | 1.55 | 0.99 | 0.4 | 0.81 | 1.41 | 1.1 | 0.79 | -1.21 | 0.83 |
| 74 | 0.49 | | 1.32 | 1.1 | 1.68 | 1.17 | 1.42 | -1.09 | -0.33 | 0.81 | 1.57 | 1.38 | 1.56 | -0.46 | 1.03 | 1.64 |
| 75 | 0.07 | | 0.98 | 1.39 | 1.65 | 1.22 | 0.58 | 1.46 | 1.72 | -0.25 | 0.03 | -0.17 | 1.15 | 1.71 | 0.09 | 2.08 |
| 76 | 0.8 | | 1.16 | 1.17 | 1.75 | 1.68 | 1.2 | -0.89 | 0.43 | 1.12 | 1.46 | 1.31 | 1.32 | -0.09 | 0.36 | 0.8 |
| 77 | 0.66 | | 1.65 | 2.18 | 2.75 | 2.03 | 1.69 | 1.68 | -0.35 | -0.66 | 0.72 | -1.13 | 1.39 | -0.33 | -0.93 | -0.51 |
| 78 | 0.05 | | 1.04 | 1.58 | 2.55 | 1.63 | 1.21 | 1.85 | 1.91 | 1.39 | 0.16 | 0.5 | 0.97 | 1.35 | 1.64 | 0.89 |
| 79 | 0.39 | | 1.5 | 1.27 | 2.3 | 1.26 | 1.76 | 2.12 | 0.78 | 0.73 | 0.84 | -0.05 | 1.27 | 2.19 | 0.63 | 1.19 |
| 80 | 0.58 | | 1.31 | 1.4 | 1.44 | 0.93 | 0.65 | -0.93 | 0.32 | 0.34 | -0.28 | 0.65 | 0.49 | -0.24 | 0.65 | 1.24 |
| 81 | 0.33 | | 1.44 | 1.35 | 2.03 | 1.55 | 0.84 | -0.18 | 0.23 | 1.73 | -1.22 | 0.03 | -0.6 | -1.5 | 0.13 | 0.75 |
| 82 | -0.03 | | 1.06 | 1.13 | 1.31 | 1.37 | 0.6 | 0.45 | -0.2 | -0.06 | -0.26 | -0.64 | -0.1 | -0.52 | 0.84 | -1.35 |
| 83 | 0.26 | | 0.79 | 1.23 | 1.47 | 1.2 | 1.3 | 0.79 | -0.15 | -0.97 | -1.03 | -0.13 | -1.16 | -0.29 | 1.14 | -1.61 |
| 84 | -0.31 | | 1.08 | 1.42 | 0.87 | 0.98 | 0.51 | 1.87 | 0.45 | 0.02 | -0.16 | 0.94 | 1.23 | 0.67 | -0.94 | -0.41 |
| 85 | -0.25 | | 1.06 | 1.37 | 1.41 | 1.39 | 0.26 | 0.15 | 0.78 | 2.1 | -0.78 | 0.2 | -0.37 | -0.87 | -1.87 | -0.12 |
| 86 | 0.14 | | 0.85 | 1.2 | 0.93 | 1.15 | 0.96 | -0.82 | -0.37 | 0.28 | 1.44 | 0.15 | 0.89 | 0.19 | 0.37 | 0.56 |
| 87 | 0.5 | | 1.28 | 1.42 | 1.6 | 1.21 | 0.85 | 1.21 | 0.57 | -0.23 | 1.3 | 0.78 | -0.21 | -0.91 | 0.11 | -0.44 |
| 88 | 0.01 | | 1.06 | 1.31 | 1.62 | 1.01 | 1.5 | -0.17 | -1.09 | 0.31 | 0.38 | 0.44 | -0.2 | -0.32 | 1.44 | 0.99 |
| 89 | 0.03 | | 1.22 | 1.03 | 1.42 | 1.16 | 0.77 | 0.84 | 0.67 | -0.81 | 1.25 | -0.14 | 0.03 | 1.15 | 0.08 | -0.16 |
| 90 | 0.71 | | 1.04 | 1.4 | 2.2 | 1.43 | 2.08 | 1.31 | -0.28 | -0.6 | 0.95 | 0.71 | 1.43 | 0.64 | 0.8 | 1.4 |
| 91 | 0.41 | | 1.44 | 1.92 | 1.34 | 2.27 | 1.63 | 0.8 | -2.49 | -0.04 | 0.19 | 1.36 | 1.35 | 0.91 | 1.15 | 0.69 |
| 92 | 0.26 | | 1.62 | 1.24 | 1.38 | 1.32 | 1.26 | -1.03 | 0.2 | 0.59 | 1.29 | 1.03 | 0.91 | 1.39 | 0.17 | 1.81 |
| 93 | 0.39 | | 1.75 | 1.43 | 1.33 | 1.75 | 0.92 | -0.65 |  | 1.65 | 1.69 | 1.78 | 2 | -0.91 | 0.22 | 1.51 |
| 94 | 0.11 | | 1.67 | 1.35 | 2.65 | 1.35 | 1.15 | 0.05 | 0.75 | 0.55 | 0.63 | 0.62 | 1.73 | 1.34 | 1.35 | 1.17 |
| 95 | 0.43 | | 1.26 | 1.25 | 1.7 | 1.56 | 1.27 | 0.58 | 1.04 | 1.21 | 1.21 | 0.25 | 1.64 | 1.28 | 0.71 | 1.23 |
| 96 | -0.3 | | 0.87 | 1.32 | 1.58 | 1.25 | 0.59 | 0.6 | 0.15 | -0.37 | 0.66 | 0.25 | 0.53 | 2.04 | 2.12 | 0.35 |
| 97 | 0.05 | | 0.72 | 1.34 | 1.2 | 0.86 | 0.73 | -1.21 | -0.36 | 1.33 | -0.14 | -0.98 | -0.94 | -1.75 | 1.55 | -0.75 |
| 98 | 0.04 | | 1.02 | 1.24 | 1.47 | 1.3 | 1.48 | 0.61 | 0.33 | -0.52 | -1.29 | -0.22 | 0.1 | 0.43 | 1.36 | 1.27 |
| 99 | -0.2 | | 0.78 | 1.27 | 1.48 | 1.17 | 1.66 | 0.4 | 0.23 | -0.47 | 0.7 | -0.78 | -0.08 | -0.22 | -0.18 | 0.08 |
| 100 | 0.62 | | 1.72 | 1.35 | 1.07 | 1.31 | 0.64 | -0.72 | -0.57 | 0.43 | 2.14 | 2.22 | 0.16 | -1.1 | -0.65 | 0.94 |
| 101 | 0.65 | | 1.45 | 1.41 | 0.71 | 0.95 | 1.25 | -0.23 | -0.48 | -0.42 | 0.29 | 0.35 | 0.13 | -1.62 | -1.41 | -0.59 |
| 102 | 0.32 | | 0.89 | 1.65 | 1.46 | 1.08 | 0.83 | -0.34 | -0.45 | -1.81 | 0.99 | 0.33 | -0.55 | -1 | 0.07 | -0.56 |

Table E. Normalized placental amino acid metabolic enzyme mRNA levels (Fisher-Yates transformed)

| **PLACENTA** | **BCATc** | **BCATm** | **GLUD** | **GLS1** | **GLUL** | **GPT2** | **GOT1** | **GOT2** |
| --- | --- | --- | --- | --- | --- | --- | --- | --- |
| 1 | -0.5 | -1.16 | 0.6 | -1.3 | 0.48 | -0.59 | -0.99 | -0.17 |
| 2 | -1.3 | -1.75 | 1.05 | -0.14 | 2.28 | -0.29 | -0.38 | -1.48 |
| 3 | -0.94 | -0.14 | -0.38 | 1.13 | 0.35 | -0.88 | -0.79 | -0.38 |
| 4 | -1.21 | -0.33 | 0.57 | -0.04 | -0.09 | 1.38 | -0.01 | 0.02 |
| 5 | -1.88 | -0.4 | 1.59 | 0.17 | -0.98 | -0.85 | -0.5 | -1.28 |
| 6 | -0.56 | -0.62 | 1.2 | 0.18 | -0.97 | 0.69 | -1.59 | 0.15 |
| 7 | 1.03 | 0.3 | -0.32 | 0.24 | -0.92 | 0.14 | -0.85 | 0.49 |
| 8 | -0.6 | -1.38 | 2.16 | -0.96 | 0.76 | -2.09 | 1.12 | -0.85 |
| 9 | 0.08 | -2.2 | 1.26 | -0.33 | 0.53 | -1.22 | -0.06 | -0.3 |
| 10 | -0.21 | -1 | -0.26 | -0.83 | -0.3 | -1.38 | 1.39 | -1.74 |
| 11 | 2.22 | 0.02 | -0.96 | 1.87 | 0.36 | 0.99 | -1.6 | 1.68 |
| 12 | 0.27 | -0.64 | -0.01 | 0.22 | 0.33 | 0.77 | -0.28 | 0.44 |
| 13 | -0.05 | -0.76 | 0.01 | 0.13 | 0.71 | -0.99 | 0.68 | -0.35 |
| 14 | 0.15 | -1.5 | 0.83 | -0.56 | 0.17 | 1.55 | 1.06 | -1.18 |
| 15 | -0.12 | -0.47 | 0.24 | -1.1 | -0.84 | -2.32 | -0.26 | 0.81 |
| 16 | -0.5 | 1.12 | 0.51 | -2.14 | -1.08 | 0.3 | 0.5 | -1.27 |
| 17 | -1.65 | -1.12 | 0.28 | -0.16 | -0.6 | -0.36 | -0.64 | -0.5 |
| 18 | -1.08 | -1.92 | 1.15 | -1.7 | 0.63 | -1.28 | 0.9 | -0.97 |
| 19 | -0.12 | -0.41 | -0.08 | 0.58 | 0.74 | 0.46 | 0.06 | -0.89 |
| 20 | -0.86 | -0.53 | 0.89 | -1.02 | -1.08 | 0.02 | 1.19 | -0.05 |
| 21 | 0.43 | -1.05 | 0.88 | -1.57 | -0.42 | 0.66 | 0.2 | -0.93 |
| 22 | -0.18 | -0.59 | 0.8 | -0.86 | -0.88 | 0.25 | 0.95 | 0.07 |
| 23 | -0.18 | -1.52 | -0.28 | 1.21 | 0.01 | -0.42 | -0.14 | 0.22 |
| 24 | -0.3 | -1.28 | 0.45 | -0.82 | 0.03 | -0.45 | 0.31 | -0.2 |
| 25 | 0.13 | -0.96 | 0.06 | -1.37 | 0.38 | -1.87 | 1.13 | 0.34 |
| 26 | 1.24 | 0.38 | -1.32 | 0.44 | -0.58 | -0.66 | 0.28 | 0.55 |
| 27 | -0.68 | 0.15 | 0.26 | 0.72 | -0.33 | -0.14 | 2.04 | -0.15 |
| 28 | -1.05 | -0.3 | 0.04 | 0.41 | -1.01 | -1.08 | 0.43 | -0.2 |
| 29 | -0.53 | 0.41 | -0.66 | -1.39 | -0.68 | -0.15 | 2.19 | -0.95 |
| 30 | 0.17 | 0.25 | -0.35 | -0.06 | 0.88 | -0.19 | -0.28 | 0.29 |
| 31 | -2.22 | 0.18 | 0.48 | -0.78 | -1.03 | 1.51 | 1.2 | 0.17 |
| 32 | 0.24 | -0.56 | 0.04 | -0.95 | -2.12 | -1.55 | -0.96 | 1.1 |
| 33 | -1.78 | -1.2 | 1.99 | -0.8 | -0.8 | -0.41 | 1.87 | -0.78 |
| 34 | -1.26 | -0.35 | 1.2 | -1.55 | 0.02 | -1.68 | 0.23 | -1.85 |
| 35 | -0.43 | -0.87 | 0.92 | 1.06 | -0.45 | 0.96 | 0.28 | -0.74 |
| 36 | -1.65 | -1.29 | 0.59 | -1.44 | -0.23 | -1.21 | 0.5 | 0.22 |
| 37 | -0.21 | -0.94 | 1.76 | -0.04 | 0.25 | 0.45 | 0.68 | -2.42 |
| 38 | -1.78 | -0.5 | 2.24 | -1.23 | -0.28 | -0.26 | 0.72 | -1.81 |
| 39 | -0.46 | 0.13 | -0.3 | 0.01 | 0.18 | -0.09 | 1.47 | 0 |
| 40 | -0.98 | 0.08 | 0.84 | 2.09 | -0.8 | -0.16 | 1.36 | -0.46 |
| 41 | -1.36 | -0.45 | 0.73 | -0.11 | 1.08 | 0.31 | -0.25 | -0.72 |
| 42 | 0.24 | 0.5 | -1.05 | 0.5 | 0.39 | -1.44 | 1.23 | 0.07 |
| 43 | 0.17 | 0.5 | 0.11 | 0.4 | -0.72 | -0.04 | 0.85 | -0.27 |
| 44 | -0.21 | 0.09 | -0.54 | 1.37 | -1.71 | 0.19 | 0.96 | 0.3 |
| 45 | 0.73 | -0.18 | 0.62 | 0.86 | -1.19 | 1.22 | -0.95 | 0.02 |
| 46 | -0.29 | -0.23 | 0.51 | -0.01 | 0.42 | -0.69 | 1.65 | -1.02 |
| 47 | 0.78 | -1.08 | -0.4 | 0.53 | -0.16 | -0.11 | -0.12 | -0.22 |
| 48 | 0.33 | -0.69 | -0.7 | 0.04 | -0.39 | -0.5 | 0.01 | -0.07 |
| 49 | -0.56 | -1.43 | 0.66 | -0.5 | 1.36 | 0.2 | -0.53 | -0.71 |
| 50 | -0.36 | -0.22 | -0.48 | -0.24 | 1.88 | 1.21 | -0.04 | -0.25 |
| 51 | 1.69 | -0.44 | -1.11 | 0.11 | -0.12 | -1.08 | 1 | 0.37 |
| 52 | 0.29 | 0.55 | -0.99 | 0.43 | -0.22 | -0.24 | -0.48 | 0.69 |
| 53 | 0.77 | 0.4 | -0.33 | 0.06 | 0.5 | 0.33 | 0.57 | -1.41 |
| 54 | -0.78 | 0.9 | -0.76 | -0.67 | 0.08 | -1.47 | 0.34 | 1.06 |
| 55 | -0.21 | -0.81 | 0.41 | -0.69 | -1.12 | -0.99 | 1.02 | 0.25 |
| 56 | 0.53 | 0.19 | -1.2 | -0.48 | 0.48 | -0.75 | 0.74 | 0.97 |
| 57 | -1.44 | 0.79 | -0.43 | 0.9 | -0.03 | -0.78 | 0.16 | -0.69 |
| 58 | -0.48 | 0.17 | -0.45 | 1.46 | 0.84 | -0.25 | -0.53 | 0.38 |
| 59 | 1.22 | 0.32 | -1.29 | 1.55 | -0.3 | 0.54 | -0.76 | 2.15 |
| 60 | 2.01 | 1.5 | -1.07 | -0.27 | -1.44 | 1.44 | -0.58 | -0.58 |
| 61 | 1.36 | 1.04 | -1.03 | 0.74 | -1.85 | 0.13 | -1.31 | 0.75 |
| 62 | 1.17 | 0.42 | -1.59 | 1.34 | -0.51 | 1.16 | -0.23 | 1.44 |
| 63 | 0.46 | 1.62 | -2.24 | 1.57 | -1.05 | 0.56 | 0.38 | -0.61 |
| 64 | 0.12 | 1.75 | -0.24 | -0.3 | -0.63 | -0.72 | 2.36 | 0.78 |
| 65 | 0.39 | 1.29 | 0.96 | -0.74 | 0.66 | 0.99 | 0.4 | -1.98 |
| 66 | 0.73 | -0.08 | 0.01 | -0.18 | 1.14 | 0.47 | -0.68 | -0.07 |
| 67 | 0.88 | 0.23 | -0.56 | 1.62 | 1.41 | 0.85 | -0.55 | -1.06 |
| 68 | -0.12 | 0.01 | 0.73 | 0.66 | 1.32 | -0.84 | -0.5 | -0.41 |
| 69 | 0.3 | 0.35 | 0.54 | 0.63 | -0.38 | 0.04 | 0.63 | -0.39 |
| 70 | -0.71 | -0.04 | -0.41 | -0.11 | -0.04 | 0.63 | -0.11 | -0.1 |
| 71 | -0.63 | 0.35 | 0.14 | -1.46 | -1.68 | -1.12 | 0.76 | 0.5 |
| 72 | 0.61 | 1.44 | -0.54 | 0.77 | 0.23 | 0.75 | -0.33 | 1.02 |
| 73 | 1.05 | 1.33 | -2.16 | 1.69 | 0.98 | 0.11 | -0.74 | 1.28 |
| 74 | 1.54 | 0.25 | -1.56 | 0.57 | 1.68 | -0.06 | -1.13 | 2.25 |
| 75 | -0.03 | 0.87 | -0.73 | 0.47 | -1.79 | -0.01 | -1.06 | 0.71 |
| 76 | 1.95 | 0.91 | -1.8 | 0.6 | 0.51 | 0.68 | -1.2 | 0.74 |
| 77 | 0.91 | 1.56 | 0.37 | 0.48 | 0.16 | 2.36 | 0.17 | 0.32 |
| 78 | 1.61 | 1.8 | -1.2 | 0.33 | 1.98 | 0.38 | 0.25 | 1.18 |
| 79 | 0.61 | 0.72 | -1.45 | -0.9 | -0.07 | -0.92 | 0.23 | 1.74 |
| 80 | 0.49 | 0.72 | -0.63 | -0.92 | -0.13 | -0.43 | -0.8 | 0.27 |
| 81 | -0.75 | 0.75 | 1.32 | 0.26 | 2.56 | -0.47 | -0.19 | -1.34 |
| 82 | -0.64 | 0.33 | 1.29 | -0.4 | 1.03 | 0.11 | -0.01 | 0.1 |
| 83 | -0.53 | -0.66 | 0.16 | -1.09 | 0.64 | 0.37 | 0.71 | 1.34 |
| 84 | 0.86 | 1.92 | 0.06 | -0.99 | -1.36 | 2.05 | -0.6 | 0.41 |
| 85 | -0.13 | -0.55 | 0.48 | 1.05 | 1.35 | -0.03 | -0.31 | -0.66 |
| 86 | 0.56 | 0.28 | -0.23 | 0.69 | -0.02 | -0.56 | -1.02 | 0.53 |
| 87 | 0.61 | -0.3 | -0.89 | -0.47 | 0.55 | -1.74 | -0.68 | 1.33 |
| 88 | 0.22 | -0.25 | -0.06 | -0.23 | 0.8 | 0.39 | -0.9 | 0.81 |
| 89 | 0.29 | 2.48 | 1.67 | -0.22 | -1.14 | 1.91 | -0.63 | -1.21 |
| 90 | 1.3 | 0.56 | -1.67 | -0.77 | 0.14 | 0.53 | -0.23 | 1.48 |
| 91 | 0.83 | 0.81 | -1.34 | 0.53 | -1.2 | 0.92 | -0.45 | 0.85 |
| 92 | 0.43 | 0.59 | -0.48 | 2.14 | 1.27 | 1.04 | -0.47 | 1.41 |
| 93 | -0.45 | 1.35 | 0.13 | 0.78 | 0.72 | 1.74 | -1.77 | 0.61 |
| 94 | 1.65 | 1.1 | -1.47 | 0.06 | -0.18 | 1.08 | -0.06 | 1.81 |
| 95 | 1.31 | 1.24 | -0.96 | 0.5 | -1.55 | 0.28 | -1.96 | 1.38 |
| 96 | -1.11 | -1.91 | 0.18 | -0.74 | 0.58 | 0.74 | -0.57 | 1.06 |
| 97 | 0 | -0.37 | 0.35 | -0.58 | 0.61 | -0.57 | -1.47 | -1.33 |
| 98 | -1.95 | -1.35 | 0.77 | 1.09 | 0.11 | 1.32 | -0.41 | -0.12 |
| 99 | -1.11 | 0.65 | 0.08 | -2.26 | -0.11 | -0.38 | -0.76 | -0.98 |
| 100 | 1.18 | 0.69 | -0.6 | 1 | 1.2 | 0.84 | -1.27 | 0.46 |
| 101 | 0.6 | 1.43 | 0.38 | 1.1 | -0.01 | 1.64 | -0.34 | -0.12 |
| 102 | 0.9 | -0.48 | 1.67 | -0.09 | 0.68 | -0.81 | -2.04 | -0.81 |
